# Phage liquid crystalline droplets form occlusive sheaths that encapsulate and protect infectious rod-shaped bacteria

**DOI:** 10.1073/pnas.1917726117

**Published:** 2020-02-18

**Authors:** Abul K. Tarafder, Andriko von Kügelgen, Adam J. Mellul, Ulrike Schulze, Dirk G. A. L. Aarts, Tanmay A. M. Bharat

**Affiliations:** ^a^Sir William Dunn School of Pathology, University of Oxford, OX1 3RE Oxford, United Kingdom;; ^b^Central Oxford Structural Microscopy and Imaging Centre, University of Oxford, OX1 3RE Oxford, United Kingdom;; ^c^Department of Chemistry, Physical and Theoretical Chemistry Laboratory, University of Oxford, OX1 3QZ Oxford, United Kingdom;; ^d^Wolfson Imaging Centre, Weatherall Institute of Molecular Medicine, University of Oxford, John Radcliffe Hospital, OX3 9DS Oxford, United Kingdom

**Keywords:** phage, antibiotic tolerance, cryo-EM, *Pseudomonas aeruginosa*, phase separation

## Abstract

The opportunistic pathogen *Pseudomonas aeruginosa* is a major cause of antibiotic-tolerant infections in humans. *P. aeruginosa* evades antibiotics in bacterial biofilms by up-regulating expression of a symbiotic filamentous inoviral prophage, Pf4. We investigated the mechanism of phage-mediated antibiotic tolerance using biochemical reconstitution combined with structural biology and high-resolution cellular imaging. We resolved electron cryomicroscopy atomic structures of Pf4 with and without its linear single-stranded DNA genome, and studied Pf4 assembly into liquid crystalline droplets using optical microscopy and electron cryotomography. By biochemically replicating conditions necessary for antibiotic protection, we found that phage liquid crystalline droplets form phase-separated occlusive compartments around rod-shaped bacteria leading to increased bacterial survival. Encapsulation by these compartments was observed even when inanimate colloidal rods were used to mimic rod-shaped bacteria, suggesting that shape and size complementarity profoundly influences the process. Filamentous inoviruses are pervasive across prokaryotes, and in particular, several Gram-negative bacterial pathogens including *Neisseria meningitidis*, *Vibrio cholerae,* and *Salmonella enterica* harbor these prophages. We propose that biophysical occlusion mediated by secreted filamentous molecules such as Pf4 may be a general strategy of bacterial survival in harsh environments.

## Significance

In this study, we investigate how phage molecules secreted by pathogenic *Pseudomonas aeruginosa* bacteria drive antibiotic tolerance by forming phase-separated liquid crystalline compartments around bacterial cells. This study spans across spatial scales, combining atomic structure determination using electron cryomicroscopy with cellular electron cryotomography, optical microscopy, and biochemical reconstitution. We show that encapsulation of rod-shaped bacteria by spindle-shaped liquid crystalline droplets made of phage molecules is a process profoundly influenced by shape and size complementarity.

*Pseudomonas aeruginosa* is an opportunistic bacterial pathogen responsible for human lung, bone, and wound infections (1). *P. aeruginosa* adapts to hostile environments by forming micrometer-scale, surface-attached communities of cells encased in an extracellular polymeric substance matrix, called biofilms (2). *P. aeruginosa* infections are difficult to clear due to increased antibiotic tolerance of the bacterial cells (3). Hence, there is an urgent need to understand fundamental mechanisms of bacterial antibiotic tolerance to develop strategies for treating *P. aeruginosa* infections.

Previous microarray studies have revealed that compared to planktonic bacteria, genes encoding prophages called Pf undergo the highest up-regulation in *P. aeruginosa* biofilms (4), indicating that Pf phages form an important part of the *P. aeruginosa* biofilm lifestyle. Pf phages also play an important role in human infections, since a large percentage of sputum samples from patients with cystic fibrosis were found to be positive for Pf phages (5). Furthermore, analysis of *P. aeruginosa* clinical isolates from chronic wound infections showed that presence of Pf in isolates correlated with the most persistent infections (6).

Pf phages promote *P. aeruginosa* survival in harsh environments by several independent mechanisms. During bacterial infections, Pf4 (a Pf phage) acts as a decoy for the host immune system by triggering an antiviral response, thus impairing bacterial clearance at the site of infection (6). Pf4 also promotes antibiotic tolerance of *P. aeruginosa* by self-assembling into higher-order liquid crystalline structures (7). These liquid crystalline structures possess a net negative electric charge due to the presence of large amounts of phage DNA, and have thus been proposed to sequester positively charged antibiotics such as tobramycin (7).

Pf4 and other Pf phages are members of the *Inoviridae* family which have recently been shown to be pervasive across all prokaryotes (8). Inoviruses are filamentous and rod-shaped, roughly six nm in diameter, that contain a single-stranded DNA (ssDNA) genome (9). The capsid structure and DNA genome topology of inoviruses has been studied by X-ray diffraction on filamentous phage containing DNA (fd) (9), Pf1 (10), and by electron cryomicroscopy (cryo-EM) on the IKe phage (11). These structural studies showed a helical array of capsid coat protein subunits surrounding a circular ssDNA genome. This genome organization in the virion has been proposed for the *Inoviridae* family (9), although an atomic-resolution confirmation of this expectation is as yet unavailable.

In this study, we investigated the molecular mechanism of Pf4-mediated antibiotic tolerance across spatial scales from atomic structures to cellular physiology, combining cryo-EM structure determination with cellular electron cryotomography (cryo-ET), optical microscopy, and biochemical reconstitution. We have solved the atomic structure of the Pf4 phage, and linked it with the process of dynamic self-assembly of the phage into phase-separated liquid crystalline droplets. By biochemically replicating the conditions necessary for antibiotic protection combined with cellular imaging, we found that phage liquid crystalline droplets form protective compartments around rod-shaped bacterial cells. Formation of these occlusive compartments is profoundly influenced by shape and size complementarity of the liquid crystals with rod-shaped bacteria, and was observed even when bacteria were replaced by inanimate colloidal rods of comparable shape and size.

There is intense interest in the cell-biology field about liquid–liquid phase separation (12). Here we present an exciting example of phase separation in bacteria with outstanding medical relevance, where we study the biophysical process bottom-up, from the level of atoms to micrometer-scale liquid crystalline droplets mediating occlusion around bacterial cells. Our data allow us to propose that biophysical occlusion may be a general strategy of bacterial survival in harsh environments.

## Results

### Cryo-EM Structure of the Pf4 Phage along with Its Linear ssDNA Genome.

**Fig. 1.**
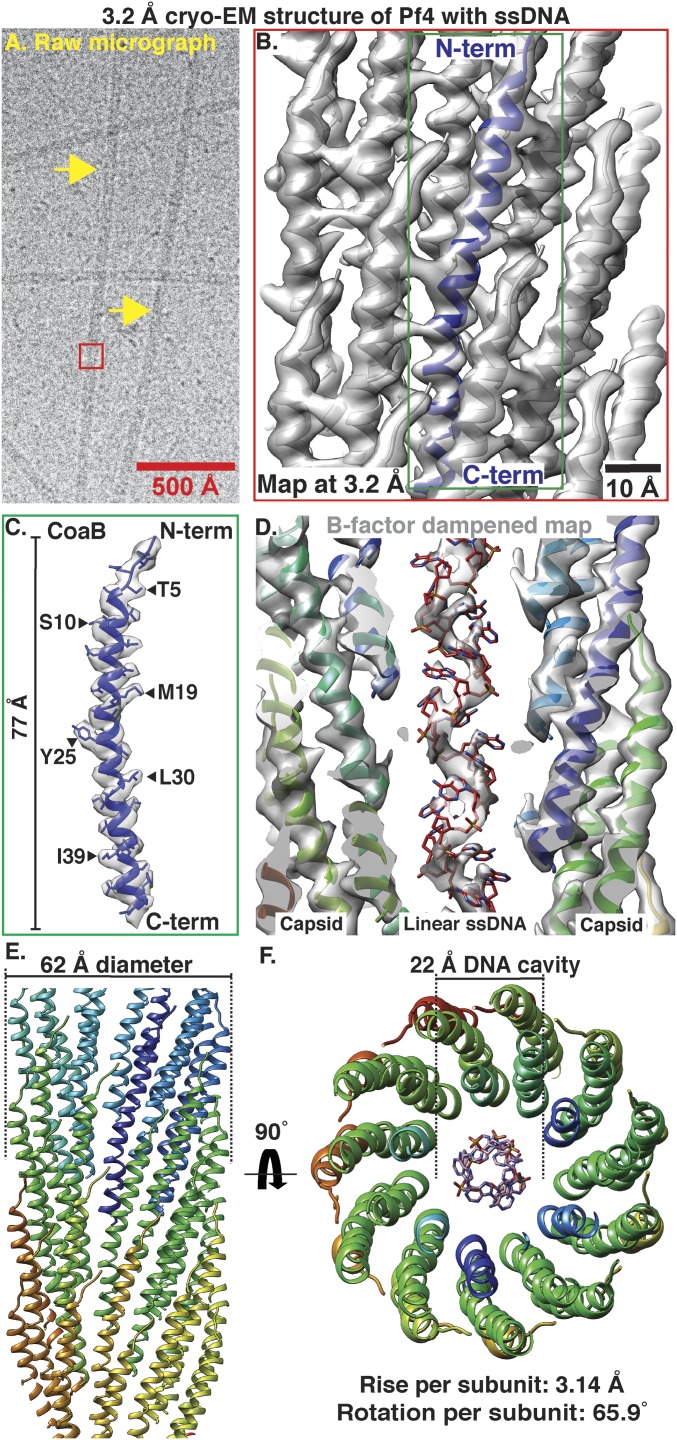
Cryo-EM structure of Pf4 phage at 3.2-Å resolution. (*A*) Cryo-EM image of native Pf4 phage (yellow arrows) purified from biofilms (red box represents reconstructed segment). (*B*) Single-particle cryo-EM reconstruction of Pf4 phage (Movie S1). Cryo-EM density is shown as a gray isosurface, with the refined atomic coordinates of Pf4 CoaB protein subunits shown as ribbons (*N*- and C terminus of one CoaB marked). (*C*) Density and atomic model for a single CoaB protein. (*D*) Cross-section through the cryo-EM structure shows that the Pf4 ssDNA genome is linear. Map is *B*-factor dampened (50 Å^2^ compared to *B* and *C*) to aid visualization of the ssDNA. (*E*) Side view of Pf4 showing interdigitated arrangement of CoaB subunits within the capsid coat (only the front CoaB subunits displayed). (*F*) Top view of Pf4 showing linear ssDNA within the 22-Å inner cavity.

We purified endogenously expressed Pf4 phage from *P. aeruginosa* PAO1 biofilms and confirmed its identity, purity, and infectivity (*SI Appendix*, Fig. S1). We imaged the purified phage using cryo-EM (*SI Appendix*, *Methods*) and observed rodlike filaments with a diameter of ∼60 Å (Fig. 1*A*), consistent with previous studies on Pf bacteriophages (10). We used real-space helical reconstruction (13, 14) to resolve a 3.2-Å resolution structure of the Pf4 phage (Fig. 1 *B*–*D*, *SI Appendix*, Fig. S2 and Table S1, and Movie S1). All side-chain densities for the 46-residue mature coat protein B (CoaB) of Pf4 are unambiguously assigned in our map (Fig. 1 *B*–*D*). The CoaB subunits adopt a 77-Å-long, α-helical structure, with the C-termini pointed toward the core of the phage and the *N*-termini directed toward the outside of the cylindrical filament (Fig. 1 *C* and *D*). Pf4 filaments show a rise and rotation per subunit of 3.14 Å and 65.90°, respectively, allowing multiple CoaB molecules to pack tightly around the filament axis in an interdigitated array (Fig. 1 *E* and *F*). The structure shows that Pf4 filaments have a diameter of ∼62 Å and an inner cavity with a diameter of ∼22 Å (Fig. 1*F*). No significant resolution anisotropy is observed in the reconstruction, indicating that CoaB forms a rigid structure in the assembled phage (*SI Appendix*, Fig. S2 *A*–*D*), consistent with previous structural studies on inoviruses (10, 11). Comparison of the Pf4 structure to the recently published cryo-EM structure of IKe, a class I phage (11, 15), shows that the major coat proteins of both phages adopt a similar elongated α-helical structure [Cα root-mean-square deviation (RMSD) of 1.5 Å] despite sharing low sequence homology (*SI Appendix*, Fig. S3 *A* and *C*). Both assembled phages have similar diameters of 62 and 64 Å, respectively, with a 22-Å cavity housing the genomic DNA (*SI Appendix*, Fig. S3*B*), however the helical symmetry is different (compare IKe phage rotation and rise 38.5° and 16.77 Å with Pf4 rotation and rise 65.9° and 3.14 Å), leading to different overall arrangement of the capsid monomers.

Density for the ssDNA genome inside the 22-Å cavity in the core of the Pf4 phage is clearly resolved (Fig. 1*D*). Unexpectedly, this density shows that the Pf4 ssDNA genome is linear, rather than circular ssDNA, which was the expected paradigm for members of the *Inoviridae* family, since it was proposed for the related bacteriophage Pf1 (10) as well as fd (9). Clear density for the phosphate backbone of the ssDNA genome is resolved (Fig. 1*D*), but no density for a second antiparallel phosphate backbone is observed, as would be expected for circular ssDNA. Although the phosphate backbone is clearly resolved, bases are smeared due to averaging along the Pf4 genome (Fig. 1*D*). The pitch of the linear ssDNA is 15.7 Å (*SI Appendix*, Fig. S2*E*), meaning that there is exactly one CoaB protein for every base of ssDNA in the phage genome. In the previous cryo-EM structure of the IKe phage, the density for single DNA bases and the phosphate backbone of the circular ssDNA genome was not resolved, but the DNA appeared to be stabilized by electrostatic interactions with positively charged arginine and lysine residues in the capsid leading to a noninteger nucleotide to protein subunit ratio of ∼2.4, as opposed to a nucleotide-to-protein ratio of exactly 1 in Pf4. Consistent with the observation of Pf4 only containing linear ssDNA in the cavity, the electrostatic potential in the cavity of Pf4 is lower than that in IKe (*SI Appendix*, Fig. S3*B*) due to a larger number of positively charged residues in the C terminus of IKe p8 that line the cavity pore (*SI Appendix*, Fig. S3*C*).

### Pf4 Phage without an ssDNA Genome Retains Filamentous Capsid Integrity.

**Fig. 2.**
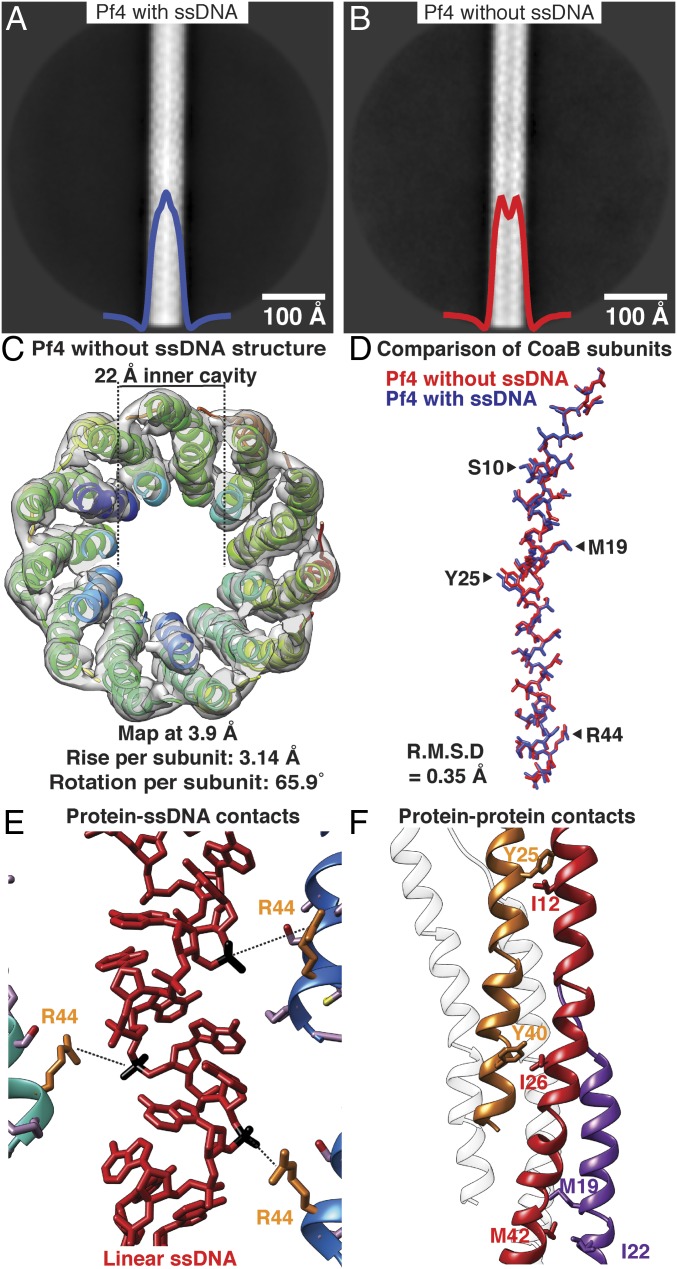
Cryo-EM structure of Pf4 filament without ssDNA at 3.9-Å resolution. (*A*) Two-dimensional class average of Pf4 with ssDNA, showing density in the core of the phage, indicated by peak in the horizontal density profile overlaid on average (blue curve). (*B*) Class average of Pf4 without ssDNA; a dip is observed in the horizontal density profile in the center of the average (red curve). (*C*) Top view of the cryo-EM structure of Pf4 without ssDNA at 3.9-Å resolution. Density is displayed as a gray isosurface and CoaB subunits as ribbons. The structure confirms lack of ssDNA in the 22-Å inner cavity. (*D*) Comparison of CoaB structure from Pf4 filaments with (blue) and without ssDNA (red) shows that the structures are almost identical (RMSD 0.35 Å, see Movie S2). (*E*) Magnified view of the CoaB:ssDNA interactions in the native Pf4 structure (from Fig. 1). Phosphates (black) of the linear ssDNA (red) are weakly coordinated by arginine 44 residues (orange) of CoaB (distance 5.4 Å). (*F*) Magnified view of CoaB:CoaB interactions within Pf4 filaments shows intersubunit hydrophobic interactions that stabilize the capsid.

In the cryo-EM dataset of native Pf4, we detected class averages in which the inner cavity of the phage appeared to be empty. We hypothesized that two compositional variants of Pf4, with or without the ssDNA genome, were present in our dataset. These were represented by class averages with a strong density in the core of the phage filament (66% of particles, Fig. 2*A*) and averages without this density in the filament core, respectively (34% of particles, Fig. 2*B*). We processed the particles from class averages with an empty inner cavity and elucidated a 3.9-Å resolution structure (Fig. 2*C*, *SI Appendix*, Fig. S4 and Table S1, and Movie S2). The structure showed clear absence of the linear ssDNA genome, confirming our hypothesis. Apart from the absence of the ssDNA genome, the overall structure of the CoaB protein coat and its packing around the filament axis (*SI Appendix*, Fig. S4*E*), which determines filament morphology, were retained. Estimated symmetry parameters for Pf4 filaments with or without ssDNA were identical, and the CoaB protein showed almost no conformational differences in the two structures (RMSD of 0.35 Å, Fig. 2*D*). This demonstrates that the ssDNA genome is not required to maintain the integrity of the Pf4 phage, contrary to what was proposed for other inoviruses (9). Analysis of the interaction between CoaB protein subunits and the linear ssDNA genome (in the Pf4 structure with ssDNA, Fig. 1) shows weak contacts between arginine 44 residues and the phosphate backbone of the DNA (Fig. 2*E*). There are no other significant protein:DNA interactions, explaining why the absence of DNA leaves the protein structure largely unaffected. Absence of a DNA genome in some filaments has not been observed in other inoviruses, and is possibly a consequence of this weak but cooperative capsid:DNA interaction. The capsid coat itself is comparatively tightly packed, and is maintained by several hydrophobic interactions between the CoaB subunits (Fig. 2*F*), which repeat along the filament, likely providing large net stability to the phage structure.

### Pf4 Phage Assembly into Liquid Crystalline Droplets Is Dynamic, with a High Degree of Orientational Ordering.

**Fig. 3.**
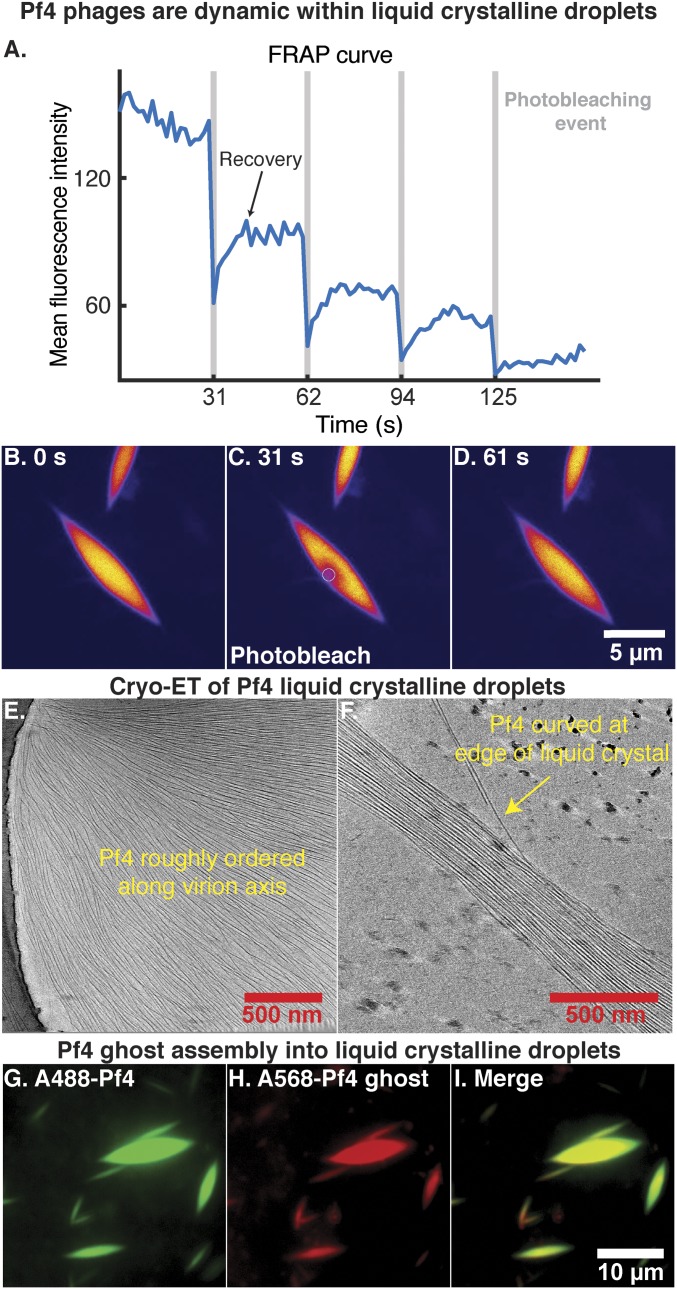
Pf4 assembly into liquid crystalline droplets is dynamic with strong orientational ordering of filaments. (*A*) A region within a liquid crystalline droplet was photobleached multiple times and the FRAP was measured over time (Movie S3). Plot shows mean fluorescence intensity of the area targeted for photobleaching (*y* axis) against time (*x* axis). Photobleaching events are indicated by vertical gray bars. (*B*–*D*) Fluorescent images at various time points during the FRAP experiment. Images have been background subtracted (yellow, high signal; blue, low signal). (Scale bar: *B*–*D*, 5 μm.) (*B*) Pf4 liquid crystalline droplet before photobleaching. (*C*) Photobleaching of a region within the Pf4 liquid crystalline droplet indicated by the white circle. (*D*) Recovery of the fluorescence signal in the photobleached region indicating that Pf4 filaments are dynamic within the liquid crystal phase. (*E* and *F*) Cryo-ET of Pf4 liquid crystalline droplets (protein density black) shows longitudinal alignment of Pf4 filaments with the axis of the spindle (Movie S4). Curved Pf4 filaments are seen at the edges of the liquid crystalline droplet. (*G*–*I*) Pf4 ghosts, with ssDNA chemically removed, can assemble into liquid crystalline droplets in the same manner as native Pf4. A488-Pf4 (green, *G*) and A568-Pf4 ghosts (red, *H*) were mixed with sodium alginate to form liquid crystalline droplets. Both compositional variants of Pf4 phage colocalized to the same liquid crystalline droplets (*I*). (Scale bar: *G*–*I*, 10 μm.)

Pf4 forms higher-order structures when mixed with biopolymers such as alginate, an anionic polysaccharide abundant in bacterial biofilms, or hyaluronan, a polymer found in human airways (7). Upon mixing Alexa-488 (A488) labeled Pf4 with sodium alginate (*SI Appendix, Supplementary Materials and Methods*), the mixture spontaneously separated into two coexisting aqueous phases: one phase rich in the biopolymer, the other phase rich in Pf4 particles. The latter phase formed droplets with a distinctive spindle shape (*SI Appendix*, Fig. S5), a result of the interplay between elastic effects, anchoring, and surface tension (16, 17). Such liquid crystalline droplets are called tactoids (7, 18). The liquid crystal phase separation was rapid, as immediately after mixing the reagents, small liquid crystalline droplets were observed (*SI Appendix*, Fig. S5*A*). These grew to over 4 µm in length (*SI Appendix*, Fig. S5 *A*–*F*), and instances where small droplets coalesced into larger droplets were observed, indicating dynamic assembly. To confirm the dynamic nature of filaments within the spindle-shaped liquid crystalline droplets, we used real-time fluorescence microscopy. A small area of the Pf4 tactoid was photobleached multiple times, and fluorescence recovery after photobleaching (FRAP) was measured (Fig. 3 *A*–*D* and Movie S3). The fluorescence signal recovered within seconds after multiple photobleaching events, proving that the individual filaments comprising the liquid crystalline droplets are dynamic within the tactoid.

To examine the ultrastructure of reconstituted liquid crystalline droplets, we applied cryo-ET to smaller tactoids that were amenable to cryo-EM, to resolve each individual phage filament within the spindle (Fig. 3*E*). In tomograms, the long axes of the Pf4 phage filaments are longitudinally aligned to the spindle axis of the liquid crystalline droplets (Movie S4). Spontaneous ordering of filaments is entropic in origin and further promoted by the biopolymer, which most likely induces an attractive depletion interaction between the filaments where the biopolymer is preferentially excluded (19). Orientational ordering, but positional randomness of Pf4 filaments are a strong indication of a nematic liquid crystalline phase (18). Filaments at the center of droplets, where the spindle is widest in diameter, are densely packed and straight, whereas filaments at the edges of spindles appear to be more curved (Fig. 3 *E* and *F* and Movies S4 and S5 *G* and *H*).

### Pf4 Liquid Crystalline Droplet Formation Is Independent of ssDNA Genome.

Since negatively charged ssDNA of Pf4 is implicated in binding positively charged antibiotics such as tobramycin (7), we examined the role of the ssDNA genome in the assembly of liquid crystalline droplets. Endogenously purified, native Pf4 is a mixed population of filaments with and without the ssDNA (Figs. 1 and 2). We produced Pf4 filaments where the ssDNA genome was extracted from the preparation, termed Pf4 ghosts, by chemical treatment with lithium chloride (20). Following DNA removal, infectivity was reduced by several orders of magnitude, while the morphology of Pf4 filaments was unaltered (*SI Appendix*, Fig. S6 *A*, *B*, and *E*), confirming the stability of Pf4 filaments without ssDNA (Fig. 2). Filament length measurements from cryo-EM images of native Pf4 reveal a population at ∼3.8 µm probably corresponding to Pf4 phages containing a complete ssDNA genome based on the calculated capsid-to-nucleotide ratio, together with the helical filament symmetry observed in our high-resolution cryo-EM structure (Fig. 1). Along with these full-length Pf4 phages, a substantial population of shorter filaments of native Pf4, presumably lacking a full ssDNA genome, were observed. Corresponding length measurements of Pf4 ghost filaments revealed that concomitant with the loss of the ssDNA genome, the average length of Pf4 ghost filaments was reduced and a filament population at 3.8 µm was not observed (*SI Appendix*, Fig. S6 *C* and *D*). Next, we tested whether Pf4 ghosts could form liquid crystalline droplets, to assess the role of ssDNA in tactoid assembly. Pf4 ghosts (*SI Appendix*, Fig. S6 *E*–*G*) labeled with Alexa-568 (A568) mixed with sodium alginate yielded spindle-shaped liquid crystalline droplets with an identical morphology (*SI Appendix*, Fig. S6 *F* and *G*) as droplets made with native Pf4 (*SI Appendix*, Fig. S6 *I* and *J*). Furthermore, A568-Pf4 ghost filaments coassembled with A488-Pf4 into the same liquid crystalline droplet (Fig. 3 *G*–*I*). This demonstrates that the negatively charged ssDNA of Pf4 is not necessary for phage assembly into liquid crystalline droplets and that filamentous rods made of the phage capsid proteins are sufficient for the formation of these higher-order tactoids.

### *P. aeruginosa* Antibiotic Protection Mediated by Pf4 Liquid Crystalline Droplets Does Not Require Negatively Charged Phage Genomic DNA.

**Fig. 4.**
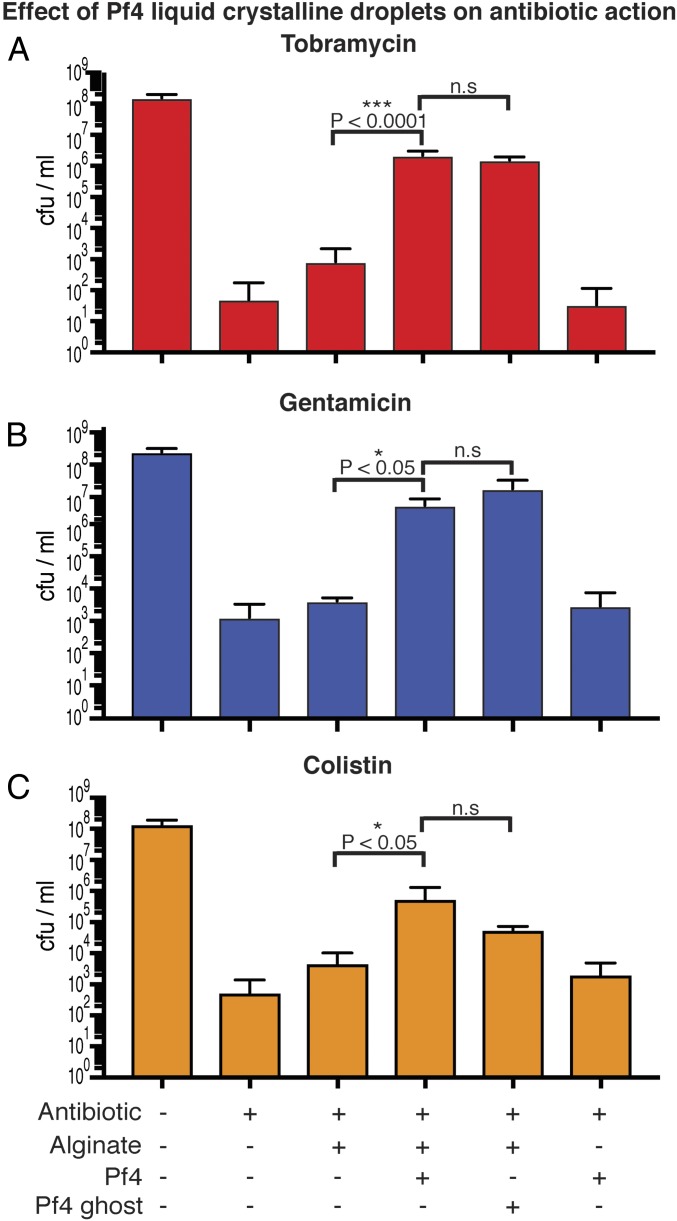
Pf4 liquid crystalline droplets with and without ssDNA protect *P. aeruginosa* cells against antibiotics. (*A*) Bar graph shows colony-forming units (cfu) per ml, a measure of *P. aeruginosa* culture cell viability after tobramycin treatment (*y* axis) in the presence of different reagents (*x* axis). Pf4 (native) and Pf4 ghost (without ssDNA) liquid crystalline droplets protect *P. aeruginosa* against tobramycin to a significantly greater extent than with sodium alginate alone (*P* < 0.0001). However, no significant difference is observed between treatments with Pf4 liquid crystalline droplets and Pf4 ghost liquid crystalline droplets. Values shown are the mean of six independent experiments (error bars show SD). (*B*) Graph shows cfu/mL (*y* axis) in the presence of different reagents (*x* axis). Pf4 liquid crystalline droplets had a significant protective effect over sodium alginate alone (*P* < 0.05) against gentamicin, but there was no significant difference between Pf4 liquid crystalline droplets and Pf4 ghost liquid crystalline droplets. (*C*) Pf4 liquid crystalline droplets had a significant protective effect over sodium alginate alone (*P* < 0.05) against colistin, but there was no significant difference between Pf4 liquid crystalline droplets and Pf4 ghost liquid crystalline droplets. Values shown are the mean of three independent experiments (error bars show SD). n.s, not significant.

Pf4 phages have been shown to promote aminoglycoside antibiotic tolerance of *P. aeruginosa* (7). It has been proposed that positively charged aminoglycoside antibiotics are sequestered by polyanions such as DNA (21). We tested this hypothesis in the laboratory, and treated cultures of PAO1 *ΔPA0728* (lacking the Pf4 integrase) with tobramycin or gentamicin either in the presence or absence of Pf4 liquid crystalline droplets (Fig. 4 *A* and *B*). Liquid crystalline droplets formed by both native Pf4 (with ssDNA) and Pf4 ghosts (without ssDNA) protected *P. aeruginosa* against tobramycin or gentamicin significantly more than the negatively charged sodium alginate alone (*P* < 0.0001 and *P* < 0.05, respectively) or Pf4 filaments alone (*P* < 0.0001 and *P* < 0.05 Fig. 4 *A* and *B*). However, there was no significant difference between the protective effect of Pf4 liquid crystalline droplets and Pf4 ghost liquid crystalline droplets for either antibiotic (Fig. 4 *A* and *B*). To determine whether this protective effect could be replicated with other classes of antibiotics, we tested colistin, an antibiotic used against *P. aeruginosa*. As seen with the aminoglycoside antibiotics tobramycin and gentamicin, both native Pf4 and Pf4 ghost liquid crystalline droplets could protect *P. aeruginosa* bacteria against colistin significantly more than the negatively charged sodium alginate alone (*P* < 0.05, Fig. 4*C*). These observations, with different antibiotics, show that the presence of liquid crystalline structures rather than negatively charged molecules such as alginate or DNA are critical for antibiotic protection of *P. aeruginosa*. To further characterize the protective effect of Pf4 liquid crystalline structures, we investigated the influence of antibiotic and Pf4 phage concentration on bacterial survival (*SI Appendix*, Fig. S7 *A* and *B*). Increasing tobramycin concentration abrogated Pf4 liquid crystalline droplet-mediated protection, indicating that the effect is dose-dependent (*SI Appendix*, Fig. S7*A*). Pf4 phage concentrations above 0.5 mg/mL (corresponding to ∼5 × 10^6^ pfu/mL, *SI Appendix*, Fig. S7*C*) were able to protect *P. aeruginosa* against tobramycin (*SI Appendix*, Fig. S7*B*). This Pf4 concentration is well below the ∼10^11^-pfu/mL reported in *P. aeruginosa* biofilms (22) suggesting protection via a Pf4-mediated mechanism would be compatible with the in vivo scenario within biofilms.

### Pf4 Liquid Crystalline Droplets Encapsulate *P. aeruginosa* Cells in a Process Profoundly Influenced by Size and Shape Complementarity.

**Fig. 5.**
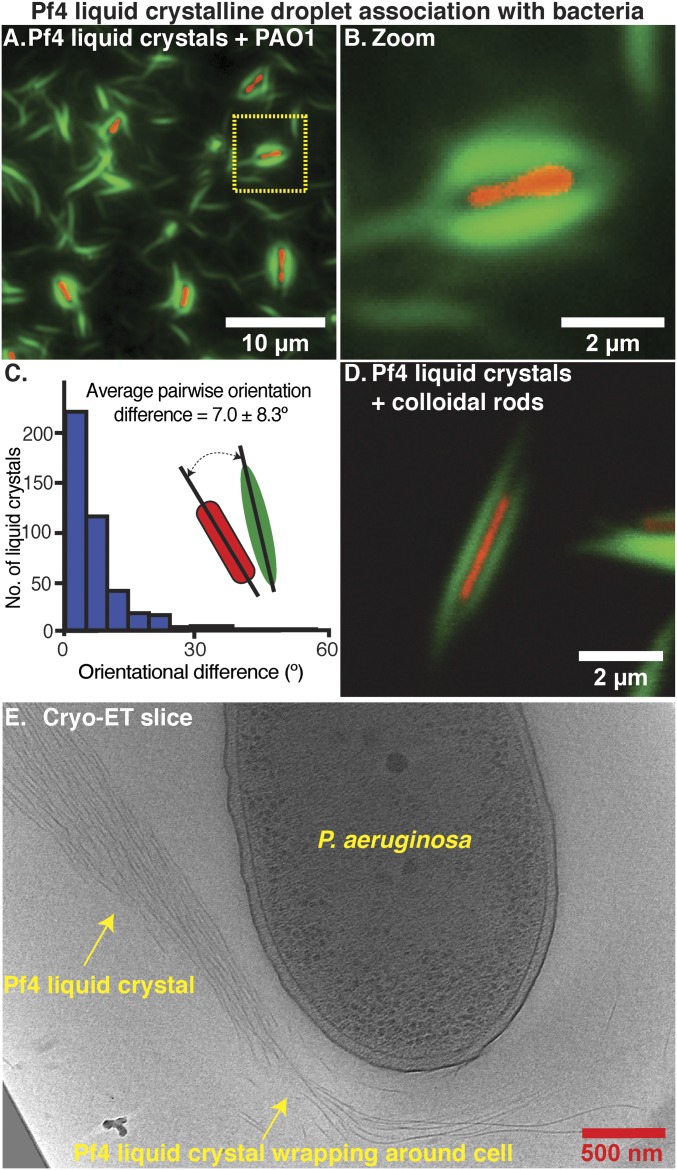
Pf4 liquid crystalline droplets form protective sheaths around *P. aeruginosa* cells. (*A*) Optical microscopy of the condition from the antibiotic protection assay presented in Fig. 4, containing *P. aeruginosa* cells, alginate, and Pf4 (Fig. 4*A*, bar 4, no antibiotic). Transmitted light channel shows bacteria (red pseudocolor) and green fluorescent channel shows Pf4 (green). (*B*) Zoom of cell shows close association of liquid crystalline droplet around the cell. (*C*) Histogram of pairwise orientational differences between bacterial cells and associated liquid crystalline droplets from automated segmentation of fluorescence images (*n* = 417). (*D*) Colloidal rods (with a similar morphology to bacteria) mixed with Pf4 liquid crystalline droplets show the same encapsulation effect. (*E*) Cryo-ET slice showing a Pf4 liquid crystal phase associated with and wrapping around a bacterial cell (Movie S5).

To study the mechanism of liquid crystalline droplet-mediated antibiotic tolerance, we examined cultures equivalent to those from the protection assay described in Fig. 4*A* (bar 4, supplemented with A488-labeled Pf4) by fluorescence microscopy. We observed that the majority of *P. aeruginosa* bacterial cells were each surrounded by a sheath made up of a Pf4 liquid crystalline droplet (Fig. 5 *A* and *B* and *SI Appendix*, Fig. S8 *A* and *B*). To quantify the morphological parameters of these assemblies, we segmented densities corresponding to liquid crystalline droplets and cells (*n* = 417) in an automated manner (*SI Appendix*, Fig. S8*C*). The pairwise orientational differences between the long axes of bacterial cells and liquid crystalline droplets were small (mean 7.0 ± 8.3°), illustrating that the association of rod-shaped cells and spindle-shaped liquid crystalline droplets is not stochastic (Fig. 5*C*). The average length of the liquid crystalline droplets around cells (4.3 ± 0.9 µm) was 1.1 µm longer than the average length of the bacterial cells (3.2 ± 1.0 µm), allowing the liquid crystalline droplets to encapsulate bacterial cells (*SI Appendix*, Fig. S8 *D* and *E*). To further characterize the encapsulation process we tested the effect of biopolymer and phage concentration (*SI Appendix*, Fig. S9). Encapsulation of *P. aeruginosa* cells was observed at alginate concentrations of 1 mg/mL and above (*SI Appendix*, Fig. S9 *A*–*C*), as well as when alginate was replaced with another biopolymer hyaluronan at concentrations of 0.5 mg/mL and above (*SI Appendix*, Fig. S9 *D*–*F*), suggesting that the crowding effect of the biopolymer rather than its chemical effect leads to liquid crystalline droplet formation and bacterial encapsulation. Titration of Pf4 concentration against a constant amount of biopolymer showed that concentrations that could effectively encapsulate *P. aeruginosa* (*SI Appendix*, Fig. S9 *G*–*I*) were able to protect *P. aeruginosa* against antibiotics (*SI Appendix*, Fig. S7*B*), indicating a correlation between encapsulation and antibiotic protection.

To probe the specificity of the encapsulation process, we tested whether Pf4 liquid crystalline droplets would associate with any rod-shaped object by replacing bacteria with inanimate colloidal rods (23). We observed similar encapsulation events using fluorescence microscopy (Fig. 5*D* and *SI Appendix*, Fig. S9 *J*–*L*), suggesting that the encapsulation process depends on the size and shape complementarity between the bacteria and liquid crystalline droplets, although the underlying biochemistry of Pf4 and *P. aeruginosa* bacteria may also play a role. Three-dimensional (3D) confocal imaging of the protection assay culture described in Fig. 4*A* (bar 4) showed that the thickness of encapsulated bacteria together with the associated liquid crystal is at least 2 µm (*SI Appendix*, Fig. S8 *F*–*H*). Such thick samples are not amenable for cryo-EM imaging due to the limited penetrative power of the electron beam. Nevertheless, we performed cryo-ET imaging of thin areas of the protection assay culture where bacteria were partially encapsulated. This showed Pf4 filaments wrapping around cells (Fig. 5*E* and Movie S5) or lined up in close proximity to the outer membrane, separated only by lipopolysaccharide and other cell surface molecules, that are known to be present in the outer membrane of Gram-negative bacteria including *P. aeruginosa* (*SI Appendix*, Fig. S8*I*) at high copy numbers (24, 25). These cryo-ET images provide snapshots of the association of Pf4 liquid crystalline droplets and the *P. aeruginosa* cell surface, indicating that Pf4 filaments can deform and wrap around rod-shaped bacterial cells to begin encapsulation.

**Fig. 6.**
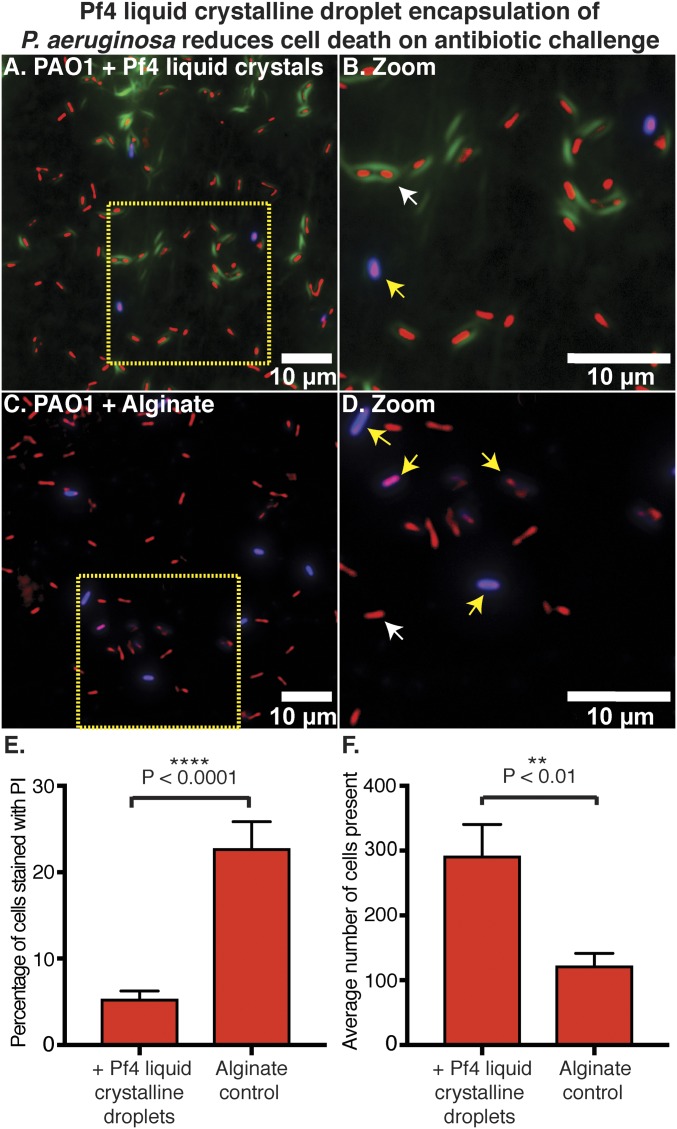
Pf4 liquid crystalline droplet encapsulation of *P. aeruginosa* prevents cell death on antibiotic treatment. (*A*) Optical microscopy of the antibiotic protection assay presented in Fig. 4, containing *P. aeruginosa* cells, alginate, Pf4, and tobramycin (Fig. 4*A*, bar 4) stained with PI. Bacteria are shown in red (pseudocolor of transmitted light channel), Pf4 liquid crystalline droplets in green, and PI staining in blue. (*B*) Zoom of *A* shows Pf4 liquid crystalline droplet encapsulated bacteria are not stained with PI (live cells indicated by white arrows and dead cells indicated by yellow arrows). (*C*) Optical microscopy of the condition lacking Pf4 filaments from the antibiotic protection assay presented in Fig. 4, containing *P. aeruginosa* cells, alginate, and tobramycin (Fig. 4*A*, bar 3) stained with PI. (*D*) Zoom of *C* shows staining of cells with PI. All fluorescence images were background subtracted. (*E*) Bar chart showing quantitation of PI staining with the percentage of cells stained with PI (*y* axis) in the presence or absence of Pf4 liquid crystalline droplets (*x* axis). (*F*) Bar chart showing the average number of cells observed per image, in randomly collected fields of the sample (*y* axis) in the presence or absence of Pf4 liquid crystalline droplets (*x* axis). Mean values are shown and the error bars denote SE. Experiment was performed in triplicate (*n* = 30 images).

To further scrutinize the link between bacterial encapsulation and antibiotic protection mediated by Pf4 liquid crystalline droplets, we examined the viability of individual cells in cultures from the protection assay in Fig. 4*A* using propidium iodide (PI) staining that allows identification of dead cells (26). Comparison of tobramycin-treated cultures containing Pf4 liquid crystalline droplets versus alginate alone showed that a significantly lower percentage of cells were stained PI positive when Pf4 liquid crystalline droplets were present (Fig. 6 *A* and *B*) as opposed to with alginate alone and in the absence of Pf4 liquid crystalline droplets (*P* < 0.0001, Fig. 6 *C* and *D*, quantitation Fig. 6*E*). Overall, a lower number of cells were observed with alginate alone (*P* < 0.01, Fig. 6*F*), suggesting increased cell death due to antibiotic action. Most importantly, PI-positive cells in images from samples containing Pf4 liquid crystalline droplets were not encapsulated (Fig. 6 *A* and *B*). Together these data show that Pf4 liquid crystalline droplet encapsulation is necessary for protection against antibiotics. One possible reason for encapsulated cells to be less susceptible to antibiotics could be that they are metabolically inactive. To test this hypothesis, we imaged Pf4 liquid crystalline droplet encapsulated *P. aeruginosa* bacteria over time using optical microscopy. We observed that encapsulated cells were able to grow and divide, suggesting they are metabolically active (*SI Appendix*, Fig. S10). Interestingly, Pf4 liquid crystalline droplets were able to rearrange to accommodate the newly divided cells reflecting their dynamic nature, confirming the observation of Pf4 filaments wrapping around *P. aeruginosa* cells observed in cryo-ET (*SI Appendix*, Fig. S10 and Movie S6).

## Discussion

Liquid–liquid phase separation has now been observed in a variety of eukaryotic systems, where it plays key roles in the formation of subcellular membraneless compartments such as nucleoli, centrosomes, and stress granules (12). Encapsulation and protection of *P. aeruginosa* by Pf4 liquid crystalline droplets is a prototypical example of phase separation in a prokaryotic system, here operating to confine entire bacterial cells. We have shown that phage liquid crystalline droplets form membraneless compartments in the presence of biopolymers, protecting bacteria from antibiotics.

**Fig. 7.**
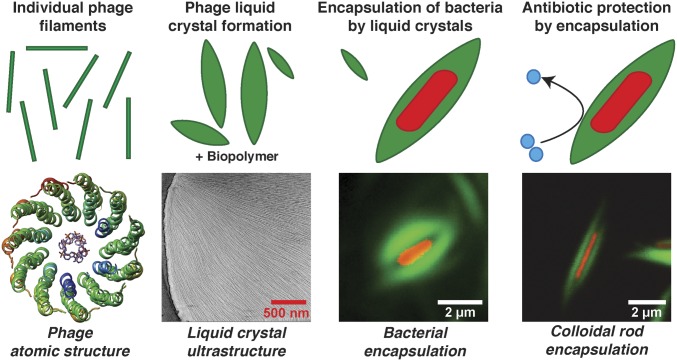
Schematic model of the mechanism of Pf4 phage-mediated antibiotic tolerance revealed in this study. Individual Pf4 phage filaments self-assemble into higher-order dynamic spindle-shaped liquid crystalline droplets termed tactoids in the presence of biopolymers. Pf4 liquid crystalline droplets encapsulate *P. aeruginosa* cells forming occlusive nanochambers that increase *P. aeruginosa* survival upon antibiotic treatment. This process is profoundly influenced by shape and size complementarity between the bacterial cells and liquid crystals as Pf4 liquid crystalline droplets can also encapsulate inanimate colloidal rods of comparable size and shape to *P. aeruginosa* bacteria.

In this study, we have probed the mechanism of Pf4 phage-mediated antibiotic tolerance across spatial scales, from the atomic structure of Pf4, to its role in forming liquid crystalline nanochambers around rod-shaped bacteria (Fig. 7). At the atomic level, although a few structures of inoviral phage capsids have been described previously (10, 11), atomic structural details about the topology of inoviral ssDNA genomes have been difficult to resolve. Based on the observation of paraxial phosphates in fiber diffraction experiments on phage Pf1, a circular ssDNA genome was proposed for all Pf phages (10). Pf4 possesses an identical capsid sequence to Pf1, and has been shown in this study to harbor a linear ssDNA genome. Given the ∼22-Å diameter of the inner cavity of Pf4 that is only weakly positive charged compared to other inoviral phages such as IKe, it is hard to envisage how a second strand of ssDNA would be accommodated inside this space in a circular ssDNA genome (Fig. 7). The linearity of the inoviral genomes, especially for integrative phages, has been suspected previously (9), and this study confirms this proposed scheme of genome arrangement. Another unexpected finding from our atomic structures was that the capsid could retain its filamentous structure in the absence of ssDNA, as the ssDNA genome was previously thought to be a scaffold, providing stability to the capsid (9).

Liquid crystalline droplets formed by rod-shaped objects have been a subject of intense inquiry in the biophysics field (18). Here in this study we have analyzed spindle-shaped liquid crystalline droplets using FRAP and high-resolution cryo-ET, and described the arrangement of Pf4 phages in the liquid crystalline phase. FRAP experiments revealed a dynamic arrangement of Pf4 within the droplet, and our cryo-ET data showed deformed Pf4 filaments at the edges of the droplets. This observation could explain the ability of the Pf4 liquid crystalline droplets to deform and encapsulate rod-shaped bacteria, forming protective nanochambers. Since Pf4 liquid crystalline droplets without negatively charged ssDNA protect *P. aeruginosa* against aminoglycosides as well as colistin (Fig. 4), our data show that electric charge-based sequestration of antibiotics (7) is not the sole basis of Pf4-mediated antibiotic tolerance. Consistent with this picture of biophysical encapsulation mediating antibiotic protection, liquid crystalline droplets were able to encapsulate inanimate colloidal rods of comparable size and shape to *P. aeruginosa* bacterial cells (Fig. 7) although the underlying biochemistry of Pf4 phage and *P. aeruginosa* might also play a role, especially within the complex environment of biofilms. PI staining together with optical microscopy demonstrated that encapsulated cells were protected against antibiotic action, but were still able to grow and divide, suggesting that metabolic inactivity is not the reason why encapsulated cells are less susceptible to antibiotics. Dividing cells were accommodated by rearrangement of the encapsulating Pf4 liquid crystalline droplets again highlighting the dynamic and fluid nature of these objects.

Recently inoviruses have been shown to be pervasive across all prokaryotes, including archaea (8). Inoviral phages similar to Pf4 are present in other pathogenic Gram-negative bacteria such as *Neisseria meningitidis*, *Vibrio cholerae*, *Klebsiella pneumoniae*, *Salmonella enterica,* and *Escherichia coli* (6, 27, 28), and their profound effect on bacterial virulence has been demonstrated (27), although the exact mechanisms remain a subject of inquiry. We suggest that biophysical occlusion of antibiotics and other harmful molecules by filamentous particles such as Pf4 or other oligomeric molecules may be a general strategy to boost bacterial survival in harsh environments.

## Methods

### Pf4 Phage Preparation.

Native Pf4 phage was initially generated from a static biofilm of a *P. aeruginosa* PAO1 strain, further amplified by infection of PAO1 on Luria-Bertani plates and isolated by polyethylene glycol precipitation as described previously (20) and in further detail in *SI Appendix*, *Materials and Methods*.

### Cryo-EM and Cryo-ET Data Collection.

Cryo-EM data of the Pf4 phage were collected as movie frame stacks using the EPU software on a Titan Krios microscope (ThermoFisher) operating at 300 kV fitted with a Quantum energy filter and a K2 Summit direct electron detector (Gatan) operating in counting mode. Tilt series data for cryo-ET were collected on the same microscope using the SerialEM software (29) in two directions starting from 0° between ±60° with 1° tilt increments. Further details on grid preparation and data collection conditions are provided in *SI Appendix*, *Materials and Methods* and Table S1.

### Cryo-EM Image Processing and Data Analysis.

Cryo-EM refinement was performed using the real-space helical reconstruction procedure implemented in Relion 3.0 (30). Fourier transforms of class averages produced using the Spring software (31), which showed high-resolution features, were indexed to determine initial helical symmetry parameters used for 3D refinement in Relion. Pf4 compositional variation was accounted for by splitting the dataset into segments from classes showing density at the core of the filament and classes where this density was absent. Model building was performed iteratively using Coot (32) along with real-space refinement against the density map in PHENIX (33). Further details are provided in *SI Appendix*, *Materials and Methods*.

### Tomogram Reconstruction from Cryo-ET Data.

Tilt series alignment using gold fiducials and tomogram reconstruction were both carried out using a set of image processing, modeling and display programs (IMOD) (34) as described previously (35). Visualization of data was performed in IMOD and UCSF Chimera (36).

### Fluorescence Microscopy and FRAP.

Samples for fluorescence microscopy were either immobilized onto agar pads constructed using Gene Frames (ThermoFisher), or applied directly to glass slides and imaged using a Zeiss AxioImager M2 widefield or Zeiss LSM Exciter confocal microscope, respectively. For FRAP experiments, samples were placed in a capillary on a glass slide and regions of interest were bleached using the Zen software bleaching mode on the Zeiss LSM exciter confocal microscope. Further details on sample preparation, imaging conditions, and image analysis are provided in *SI Appendix*, *Materials and Methods*.

### Materials and Data Availability.

Cryo-EM maps reported in this study have been deposited at the Electron Microscopy Data Bank with accession codes EMD-10593 and EMD-10594. Atomic models have been deposited in the Protein Data Bank with PDB ID codes 6TUP and 6TUQ. Reagents generated in this study will be made available on request, but we may require a payment and/or a completed Materials Transfer Agreement if there is potential for commercial application.

## Supplementary Material

Supplementary File

Supplementary File

Supplementary File

Supplementary File

Supplementary File

Supplementary File

Supplementary File
