# Supplementary material for: Phage liquid crystalline droplets form occlusive sheaths that encapsulate and protect infectious rod-shaped bacteria
Source: Proc Natl Acad Sci U S A. 2020 Feb 18;117(9):4724–31. doi: 10.1073/pnas.1917726117 (PMC7060675; doi:10.1073/pnas.1917726117)
Supplement: Supplementary File [file pnas.1917726117.sapp.pdf]

PNAS
www.pnas.org

Supplementary Information for

Phage liquid crystalline droplets form occlusive sheaths that encapsulate and protect infectious rod-shaped bacteria

Abul K. Tarafder[a,b], Andriko von Kügelgen[a,b], Adam J. Mellul[c], Ulrike Schulze[d], Dirk G.A.L. Aarts[c], and Tanmay A.M. Bharat[a,b,1]

a - Sir William Dunn School of Pathology, University of Oxford, South Parks Road, Oxford OX1 3RE, United Kingdom

b - Central Oxford Structural Microscopy and Imaging Centre, South Parks Road, Oxford OX1 3RE, United Kingdom

c - Department of Chemistry, Physical and Theoretical Chemistry Laboratory, University of Oxford, South Parks Road, Oxford OX1 3QZ, United Kingdom

d – Wolfson Imaging Centre, Weatherall Institute of Molecular Medicine, University of Oxford, John Radcliffe Hospital, Oxford OX3 9DS, United Kingdom

1 – Corresponding author

Tanmay A.M. Bharat, email: tanmay.bharat@path.ox.ac.uk

This PDF file includes:

Supplementary Materials and Methods
Figures S1 to S10
Table S1
Legends for Movies S1 to S6
SI References

Other supplementary materials for this manuscript include the following:

Movies S1 to S6

Supplementary Materials and Methods

Bacterial strains and growth conditions

P. aeruginosa strains PAO1 or PAO1 *ΔPA0728* (a kind gift from Prof. Patrick Secor, University of Montana) were used for all experiments as indicated. Unless specified otherwise, all shaking cultures of bacteria were grown at 37°C in Luria-Bertani (LB) medium with agitation at 180 rpm (revolutions per minute).

Pf4 phage production and purification

PAO1 static biofilms were grown in 100 ml LB broth at 37°C with 50 ml media exchanged every 24 hours until formation of small colony variants (SCVs) was observed. Formation of SCVs was monitored by dipping an inoculating loop into the biofilm culture and streaking onto a LB plate. Once SCVs had formed the culture was centrifuged (5,000 *g*, 15 minutes, 4°C) and the supernatant collected and passed through a 0.22 μm filter, to remove the residual bacteria. Pf4 was precipitated from the supernatant by polyethylene glycol (PEG) precipitation as described previously (1). Briefly, the supernatant solution was adjusted to 0.5 M NaCl and Pf4 precipitated by incubation with 10% (w/v) PEG 6000 (Sigma) overnight at 4°C. Resuspended phage particles were pelleted by centrifugation (12,000 *g*, 30 minutes, 4°C), resuspended again in phosphate buffered saline (PBS) solution and dialysed overnight against PBS using 10 kDa MWCO (molecular weight cut-off) snakeskin dialysis membranes (ThermoFisher). For further amplification of Pf4, 1 x 10^3 pfu (plaque forming units)/ml of the Pf4 isolated from PAO1 biofilms were incubated with 1 ml of PAO1 culture at 0.5 OD_{600} for 15 mins and mixed with hand-hot 0.8% (w/v) agar. The mix was plated onto 10 cm^2 LB-agar plates and incubated overnight at 37°C. Each plate was covered with 5 ml PBS and incubated for 6 hrs before the PBS was collected, centrifuged (12,000 *g*, 30 minutes, 4°C) and the supernatant subjected to PEG precipitation as described above. Yield of the Pf4 phage preparation was estimated and assayed using Nanodrop (Thermo Scientific) and infectivity (pfu/ml) quantified using a plaque assay as described previously (2). Presence of Pf4 in

purified preparations was confirmed by the amplification of an 839 base pair region corresponding to the replicative form of Pf4 using the following primers: **Pf4F** 5'-ATTGCTTCATCGCGCTGCT-3' and **Pf4R** 5'-TCCAGTCACAAATGGCCTCTA-3' (3). The phage was further analysed using SDS-PAGE, and the band corresponding to CoaB verified using peptide fingerprinting mass spectrometry.

Cryo-EM grid preparation

Samples for cryo-EM were prepared by pipetting 2.5 μl of the sample onto freshly glow-discharged Quantifoil grids (Cu/Rh R2/2, 200 mesh or Au R1.2/1.3, 300 mesh for helical reconstruction of Pf4 phage, Cu/Rh R3.5/1, 200 mesh for cryo-ET) and plunge frozen into liquid ethane in a Vitrobot Mark IV (ThermoFisher). For cryo-ET only, 7 μl of sample was mixed with 1 μl of protein-A conjugated with 10 nm colloidal gold (CMC, Utrecht) before 2.5 μl was applied to the grid. Plunge-frozen grids were transferred to liquid nitrogen and stored until imaging.

Cryo-EM and cryo-ET data collection

Cryo-EM data for screening specimens were collected either using a Titan Krios or Talos Arctica microscope (ThermoFisher) operated at 300 kV or 200 kV respectively. High-throughput data was collected using the EPU software on a Titan Krios microscope fitted with a Quantum energy filter (slit width 20 eV) and a K2 Summit direct electron director (Gatan) operating in counting mode at an unbinned, calibrated pixel size of 1.38 Å. A combined total dose of approximately 43 e^{-}/A^{2} was applied with each exposure lasting 10 s and 40 frames were recorded per movie. In total 4110 movies were collected between -1 to -3 μm defoci. Tilt series data for cryo-ET was collected on the same Titan Krios using the Quantum energy filter and K2 direct electron director with the SerialEM software (4). Tilt series were collected in two directions starting from 0° between ±60° with 1° tilt increment between -6 to -10 μm defoci with a combined dose of approximately 120 $e^{-}/Å^{2}$ applied over the entire series. Tilt series

were collected at an unbinned, calibrated pixel size of 5.57 Å (Figs. 3E and 5E and Fig. S8I, and Movies S3 and S5) or 4.41 Å (Fig. 3F and Fig. S5G-H).

Cryo-EM Image processing and data analysis

Real-space helical reconstruction: Data analysis was performed by following the helical reconstruction procedure implemented in Relion 3.0 (5). Movies were motion corrected and dose weighted with MotionCor2 (6). Contrast transfer functions (CTFs) of the resulting motion corrected micrographs were estimated using CTFFIND4 (7). Micrographs showing clear Thon rings visible upto at least ~6 Å were retained. Pf4 filaments were autopicked from micrographs and segments extracted along the filament with a 400 pixel x 400 pixel box. Segments were classified using reference-free 2D-classification inside Relion 3.0 and segments from classes showing high-resolution features were retained for further analysis (280,483 segments). To account for compositional variation of Pf4 (filaments with and without ssDNA), the dataset was split into segments from classes showing density at the core of the filament (185,002 segments - Pf4 with ssDNA) and those where the density at the core of the filament was absent (95,481 segments - Pf4 without ssDNA) (Fig. 2A-B and Table S1). Fourier transforms of class averages showing high resolution features were indexed to determine initial helical symmetry parameters (8). These parameters were used for 3D refinement in Relion 3.0. The final volumes were *B*-factor compensated and filtered to the obtained resolutions (Table S1). Resolutions of the structures were estimated according to the gold standard Fourier shell correlation (FSC) criterion of 0.143 (9). Visualisation of densities was carried out in UCSF Chimera (10).

Model building and refinement: The backbone of the coat protein B (CoaB) of Pf4 bacteriophage was manually traced through a single subunit of the *B*-factor sharpened cryo-EM density using Coot (11). Residues 1-46 of the mature CoaB protein were assigned by fitting side chains corresponding to bulky amino acid residues into the cryo-EM density. The full viral capsid with multiple copies of the CoaB protein around the filament was generated by applying the filament symmetry to individual subunits using pdbset in CCP4 (12). The entire

capsid was fitted using real-space refinement (13) in PHENIX (14) using non-crystallographic symmetry (NCS) restraints. Density within the inner cavity of Pf4 filaments with ssDNA map could not be explained by any residue of the CoaB protein and was therefore assigned to correspond to Pf4 genomic DNA. While the ribose phosphate backbone corresponding to a linear single-stranded genome was clearly visible, the bases of the nucleotides were smeared due to averaging over the Pf4 genome. Therefore, we built a single stranded poly-adenine chain into a *B*-factor dampened (50 Å^2) cryo-EM density using Coot and refined the structure by iteratively rebuilding the model and real-space refinement in PHENIX. Comprehensive model validation of the Pf4 with ssDNA and Pf4 without ssDNA structures were performed in PHENIX (Table S1). Figures containing protein structures or cryo-EM data were prepared using USCF Chimera.

<u>Fluorescent labelling of Pf4 phage</u>

Purified Pf4 phage was dialysed into 10 mM sodium carbonate buffer pH 9.2 using a 10 kDa MWCO snakeskin dialysis membrane (ThermoFisher). One ml of Pf4 phage (5 mg/ml) was incubated with 100 µg A488 fluorescent dye (ThermoFisher) for 1 hour at room temperature (RT) with end-over-end agitation. The sample was then passed over two PD10 desalting columns (GE Healthcare) to separate A488-labelled Pf4 phage from free A488 dye. Pf4 ghost filaments were labelled with A568 following the same protocol.

<u>Fluorescence Recovery after Photobleaching (FRAP) of Pf4 liquid crystalline droplets</u>

To carry out FRAP experiments, 3 mg/ml Pf4 and 10 mg/ml hyaluronan were mixed in a 1:1 (v/v) ratio and placed in a capillary on a glass slide. The Zeiss ZEN software bleaching mode was used on the Zeiss LSM Exciter confocal microscope. A custom circular region within the field of view was selected. A laser power of 50% was used for the photobleaching step, set to occur every 20 frames beginning at frame 20, and a laser power of 1% was used for imaging of all frames between these steps. The Pf4 liquid crystalline droplets selected for FRAP

experiments were in the bulk of the sample and imaged 2 hours after their formation. Frames were recorded with a time interval of 1.5 s.

Pf4 ghost production

The DNA genome of Pf4 was extracted using a modified version of a previously described protocol (1). Briefly, purified Pf4 phage (5 mg/ml) was incubated with 10 M LiCl in a 1:1 (v/v) ratio for 2 days at 46°C. The sample was subsequently diluted 1 in 10 with PBS and incubated with 10 µg/ml DNaseI (Sigma) and 1 U/ml benzonase (Sigma) for 2 hours at 37°C. The sample was then centrifuged (100,000 *g*, 1 hour, 4°C) and the pellet resuspended in PBS. Phage protein concentration was determined using a Nanodrop and infectivity (pfu/ml) was measured using a plaque assay as described above.

Antibiotic protection assay

An overnight culture of PAO1 *ΔPA0728* was grown in LB media at 37°C, diluted 1 in 100 into LB medium and grown at 37°C to an OD_{600} of 0.5. Hundred µl of the resulting culture was added to a 96-well plate and grown for a further 30 minutes at 37°C. Hundred µl of the indicated Pf4 and/or polymer components were added to the culture such that final concentrations of components were: sodium alginate (4 mg/ml), Pf4 (1 mg/ml) and Pf4 ghost (1 mg/ml). Additionally, 20 µg tobramycin (Sigma) (Fig. 4A) or 20 µg gentamicin (Sigma) (Fig. 4B) or 10 µg colistin (Sigma) (Fig. 4C) was added as indicated. Cultures were grown for a further 3 hours before a 10 µl sample for each assay condition was taken, serially diluted 10-fold and dilutions plated onto LB agar plates. Plates were incubated overnight at 37°C and colonies forming units (cfu) enumerated. For antibiotic titration experiments, tobramycin concentration was varied as indicated while other components of the assay remained constant as described (Fig. S7A). For Pf4 phage titration experiments, Pf4 concentration was varied as indicated in the presence of 20 µg tobramycin while other components of the assay remained constant as described above (Fig. S7B). Mean cfu/ml with standard deviation were calculated and plotted using Prism GraphPad software.

Fluorescence microscopy

Pf4 liquid crystalline droplets: 2 mg/ml A488-labelled Pf4 phage or A568-labelled Pf4 ghost was mixed in a 1:1 (v/v) ratio with sodium alginate (10 mg/ml) or hyaluronan (10 mg/ml) and incubated at room temperature for the indicated time period. Ten µl of the resulting sample was pipetted onto 0.7% (w/v) agar pads constructed using 15 x 16 mm Gene Frames (ThermoFisher) following the manufacturer's protocol, with a coverslip placed on top. The slide was imaged with a 100x objective using a Zeiss Axioimager M2 (Carl Zeiss) in bright field and fluorescence mode. Quantification of Pf4 liquid crystalline droplet major axis length was performed by thresholding, creating a bounding rectangle and measuring lengths using Fiji (15). Graphs were plotted using Prism GraphPad software. Images were background subtracted and figure panels prepared using Fiji.

Pf4 liquid crystalline droplets / P. aeruginosa bacteria: In order to image conditions equivalent to those utilised in the antibiotic protection assay, PAO1 *ΔPA0728* was grown to an OD_{600} of 0.5 and incubated with A488-labelled Pf4 (final concentration 1 mg/ml) and sodium alginate (final concentration 4 mg/ml) for 3 hours. Ten µl of the sample was pipetted onto 0.7% (w/v) agar pads constructed using 15 x 16 mm Gene Frames (ThermoFisher) with a coverslip applied, and imaged with a 100x objective using a Zeiss Axioimager M2 (Carl Zeiss). For biopolymer titration experiments, sodium alginate concentration was varied as indicated (Fig. S9A-C) or sodium alginate was replaced by high molecular weight hyaluronan (R & D Systems) at the indicated concentrations (Fig. S9D-F) while other components remained constant as described above. For Pf4 phage concentration experiments, Pf4 concentration was varied as indicated while other components remained constant as described above (Fig. S9G-I) Images were background subtracted and figure panels prepared using Fiji.

3D confocal and time-lapse microscopy of Pf4 liquid crystalline droplets / P. aeruginosa bacteria: Samples equivalent to those utilised in the protection assay were prepared as described above and z-stacks were acquired in the fluorescent and brightfield channels with an Olympus Fluoview FV1000 microscope equipped with a 100x objective. For time-lapse experiments images were acquired in the fluorescent and brightfield channels at 10 minute

intervals over a 90 minute time period at room temperature using the same microscope and objective. Time-lapse images were drift corrected using the StackReg plugin in Fiji.

Propidium iodide staining of Pf4 liquid crystalline droplets / P. aeruginosa bacteria: PAO1 *ΔPA0728* was grown to an OD_{600} of 0.5 and incubated with A488-labelled Pf4 (final concentration 1 mg/ml) and sodium alginate (final concentration 4 mg/ml) for 3 hours in the presence of 10 µg tobramycin before incubation with 0.1 mM propidium iodide for 30 minutes at room temperature. 10 µl of the sample was pipetted onto agar pads as described above and imaged with a 100x objective using a Zeiss Axioimager M2 (Carl Zeiss) in bright field and fluorescence mode. Quantitation of cell numbers and propidium iodide positive cells was performed by thresholding using Fiji. Graphs were plotted using Prism GraphPad software.

Pf4 liquid crystalline droplets/SU-8 rods: Cyanine 3 labelled SU-8 colloidal rods, fluorescent at 532 nm, were used as particles to mimic bacterial cells. These were approximately 0.5 µm in diameter and ranged between 1 and 8 µm in length. Initially dispersed in water, samples were placed in an oven for 5 minutes to dry out, before 10 mg/ml sodium alginate in PBS was added as the new solvent for the rods. Equal volumes of this solution and 3 mg/ml A488-Pf4 in PBS were combined and mixed on a vortex mixer for 5 minutes to ensure even dispersion of rods, before being placed in a capillary. This was placed onto a glass slide and imaged on the Zeiss LSM Exciter confocal microscope. A488-Pf4 tactoids were observed in one channel, and the rods were observed in the other channel (as reflected light).

<u>Automated segmentation of images with liquid crystalline droplets surrounding bacterial cells</u>

Brightfield channel images were Gaussian filtered, background subtracted and thresholded to identify the positions of bacterial cells. Bacterial cell shapes were found using the activecontour algorithm in Matlab (16). The regions of identified bacteria were dilated and used as seed inputs for the segmentation of the liquid crystalline droplets in the green channel using the activecontour algorithm. The segmented bacterial cells from the bright field and the liquid crystalline droplets from the green channel were used to calculate morphological parameters (Fig. 5 and Fig. S8).

Statistical analysis

Statistical analysis was performed using Prism GraphPad software and an unpaired t-test was used to calculate p-values.

Supplementary Figures

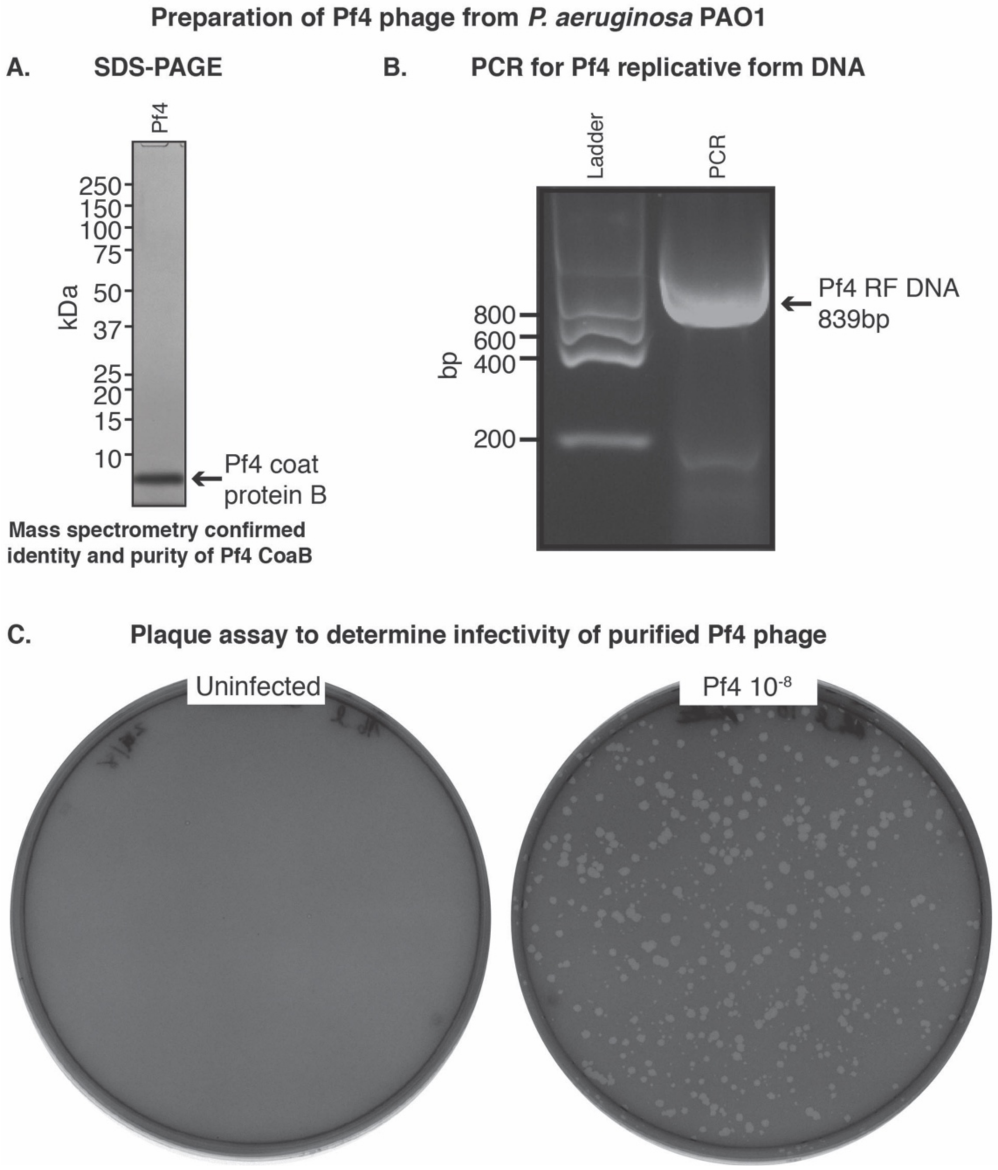

Fig. S1. Purification of Pf4 phage from *P. aeruginosa* PAO1 biofilms (related to Fig. 1)

(A) SDS-PAGE followed by Coomassie staining of endogenous Pf4 preparation shows a band corresponding to the expected size of mature Pf4 CoaB protein. Peptide fingerprint mass spectrometry of sample verified identity of band as CoaB. (B) PCR amplification with primers specific for the replicative form of Pf4 shows a fragment of the expected size indicating presence of Pf4 DNA in the specimen. (C) Plaque assay to measure infectivity of purified Pf4 on PAO1. Left, PAO1 treated with PBS (mock) shows uninterrupted lawn of PAO1 cells. Right, PAO1 infected with a 10^{-8} dilution of Pf4 shows multiple plaques in the lawn of PAO1, indicating the presence of infectious Pf4 phage.

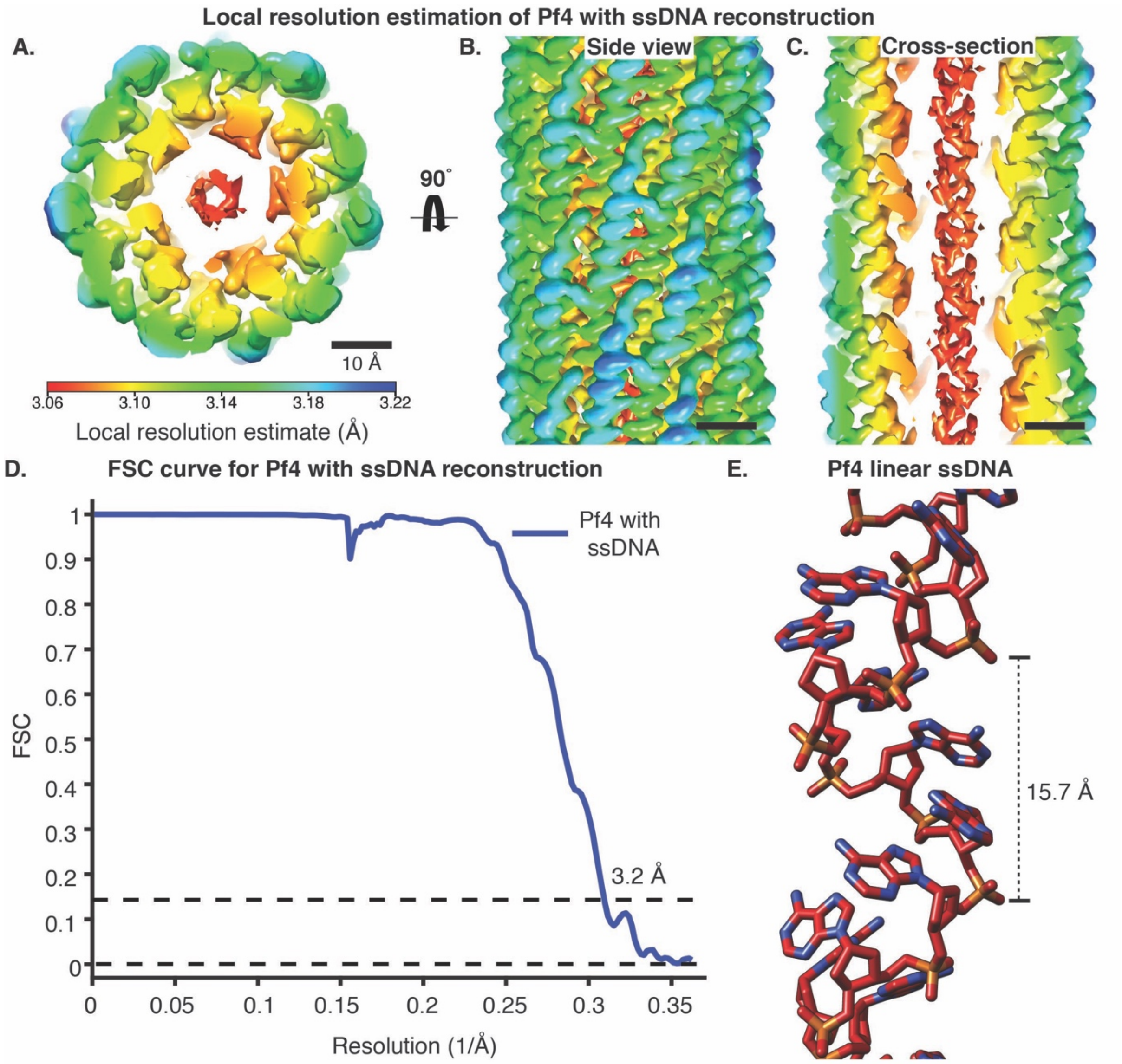

Local resolution estimation of Pf4 with ssDNA reconstruction
A.
10 Å
3.06
3.10
3.14
3.18
3.22
Local resolution estimate (Å)
90°
B.
Side view
C.
Cross-section
D.
FSC curve for Pf4 with ssDNA reconstruction
FSC
Pf4 with ssDNA
3.2 Å
Resolution (1/Å)
0
0.05
0.1
0.15
0.2
0.25
0.3
0.35
0.1
0.2
0.3
0.4
0.5
0.6
0.7
0.8
0.9
1
E.
Pf4 linear ssDNA
15.7 Å

Fig. S2. Characteristics of the Pf4 with ssDNA cryo-EM structure (related to Fig. 1)

(A) Resolution anisotropy of the Pf4 with ssDNA reconstruction (red - high resolution, blue - lower resolution). A top view is shown, filament axis going into the plane of paper. (B) Side view of the same map as in panel A. (C) Cross-section through panel B. No significant resolution anisotropy was detected in the reconstruction although CoaB side chains at the outer surface were slightly less well-resolved than those in the core of the phage. The phosphate backbone of the linear ssDNA is well-resolved but the bases are smeared due to averaging over the Pf4 genome. (D) Fourier shell correlation (FSC) curve for the Pf4 with ssDNA structure with the resolution estimated using the 0.143 criterion. (E) Magnified view of the model of Pf4 ssDNA genome. Due to smearing of bases over the Pf4 genome, poly-adenines are built into the map. The pitch of the ssDNA helix is 15.7 Å, which corresponds to a ssDNA base to CoaB subunit ratio of exactly 1.

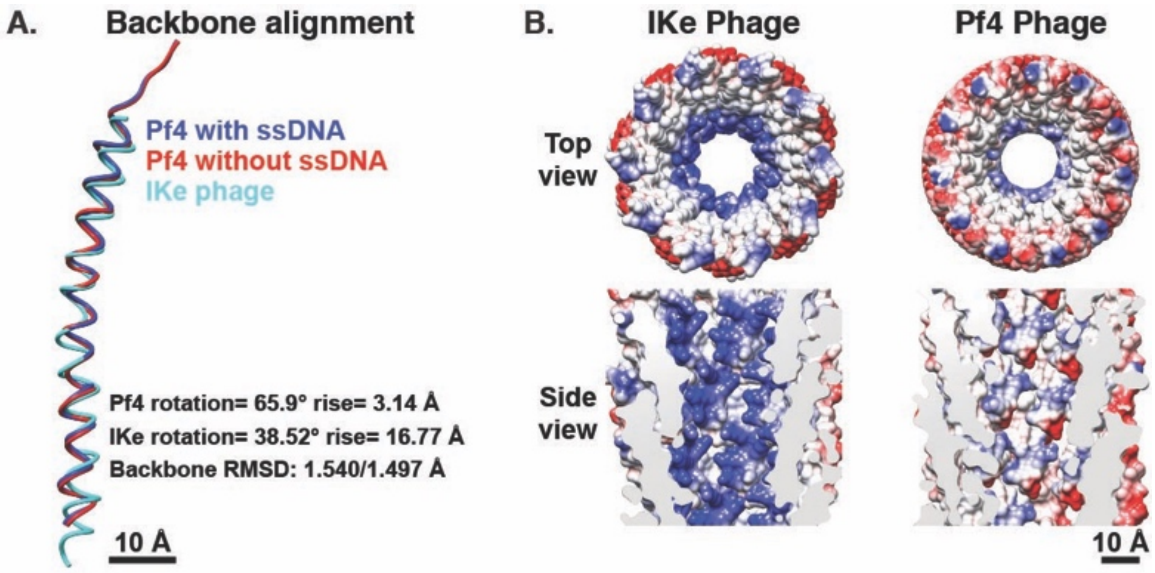

C.

```
Pf4|37-82  --GVIDTSAVESAITDGQGDMKAIGGYIVGALVILAVAGLIYSMLRKA-----
IKe|30-82  AEPNAATNYATEAMDSLKTQAIDLISQTWPVVTTVVVAGLVIRLFKKFSSKAV
If1|24-74  --ADDATSQAKAAFDSLTAQATEMSGYAWALVVLVVGATVGIKLFKKFVSRAS
Fd|24-73   ---AEGDDPAKAAFDSLQASATEYIGYAWAMVVVIVGATIGIKLFKKFTSKAS
```

Fig. S3 Comparison of Pf4 and IKe cryo-EM structures (related to Figs. 1 and 2)

(A) Comparison of major coat proteins structure from Pf4 with ssDNA (blue), Pf4 without ssDNA (red) and IKe (cyan)(17). The coat proteins have a similar α-helical structure (Cα RMSD of 1.54 Å and 1.49 Å between Pf4 with and without ssDNA and IKe) but adopt different overall helical symmetries to assemble the phage filament. (B) Top view and cross-section of IKe filament (left) and Pf4 filament (right). Electrostatic potential is plotted on the protein surfaces on a blue (positive electrostatic potential) to red (negative electrostatic potential) scale. The inner cavity of IKe is more positively charged than the Pf4 cavity. (C) Multiple sequence alignment of major coat proteins of Pf4, IKe, If1 and Fd shows low sequence homology between Pf4 and other class I phages (18). Positively charged residues in the C-termini of the major coat proteins are highlighted in blue.

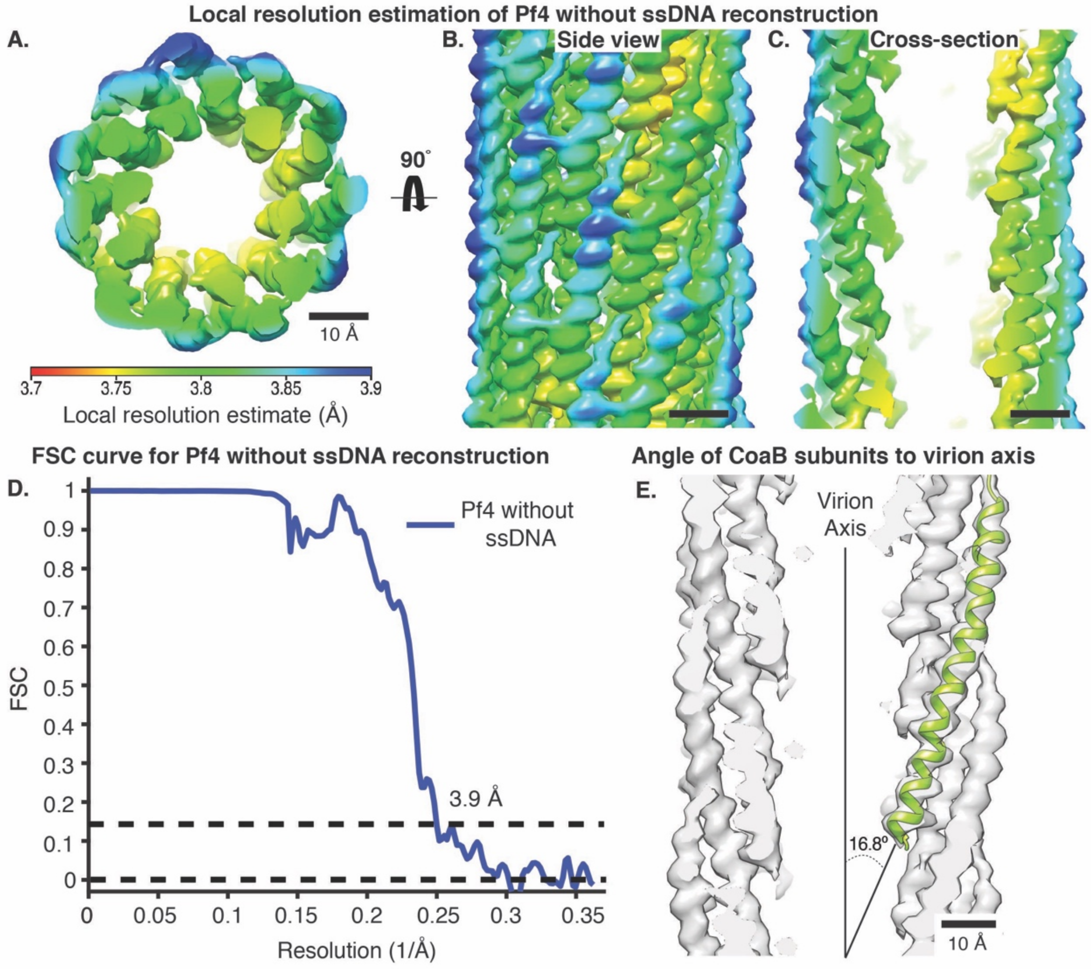
Local resolution estimation of Pf4 without ssDNA reconstruction
A.
B. Side view
C. Cross-section
90˚
10 Å
3.7
3.75
3.8
3.85
3.9
Local resolution estimate (Å)
FSC curve for Pf4 without ssDNA reconstruction
D.
Pf4 without ssDNA
3.9 Å
FSC
Resolution (1/Å)
Angle of CoaB subunits to virion axis
E.
Virion Axis
16.8º
10 Å

Fig. S4. Characteristics of the Pf4 without ssDNA cryo-EM structure (related to Fig. 2)

(A) Resolution anisotropy of the Pf4 without ssDNA reconstruction (red - high resolution, blue - lower resolution). A top view is shown, filament axis going into the plane of paper. (B) Side view of the same map as in panel A. (C) Cross-section through panel B. No significant resolution anisotropy was detected in the reconstruction. (D) FSC curve for the Pf4 without ssDNA structure with the resolution estimated using the 0.143 criterion. (E) Inclination of the CoaB protein subunits from the filament axis is 16.8º.

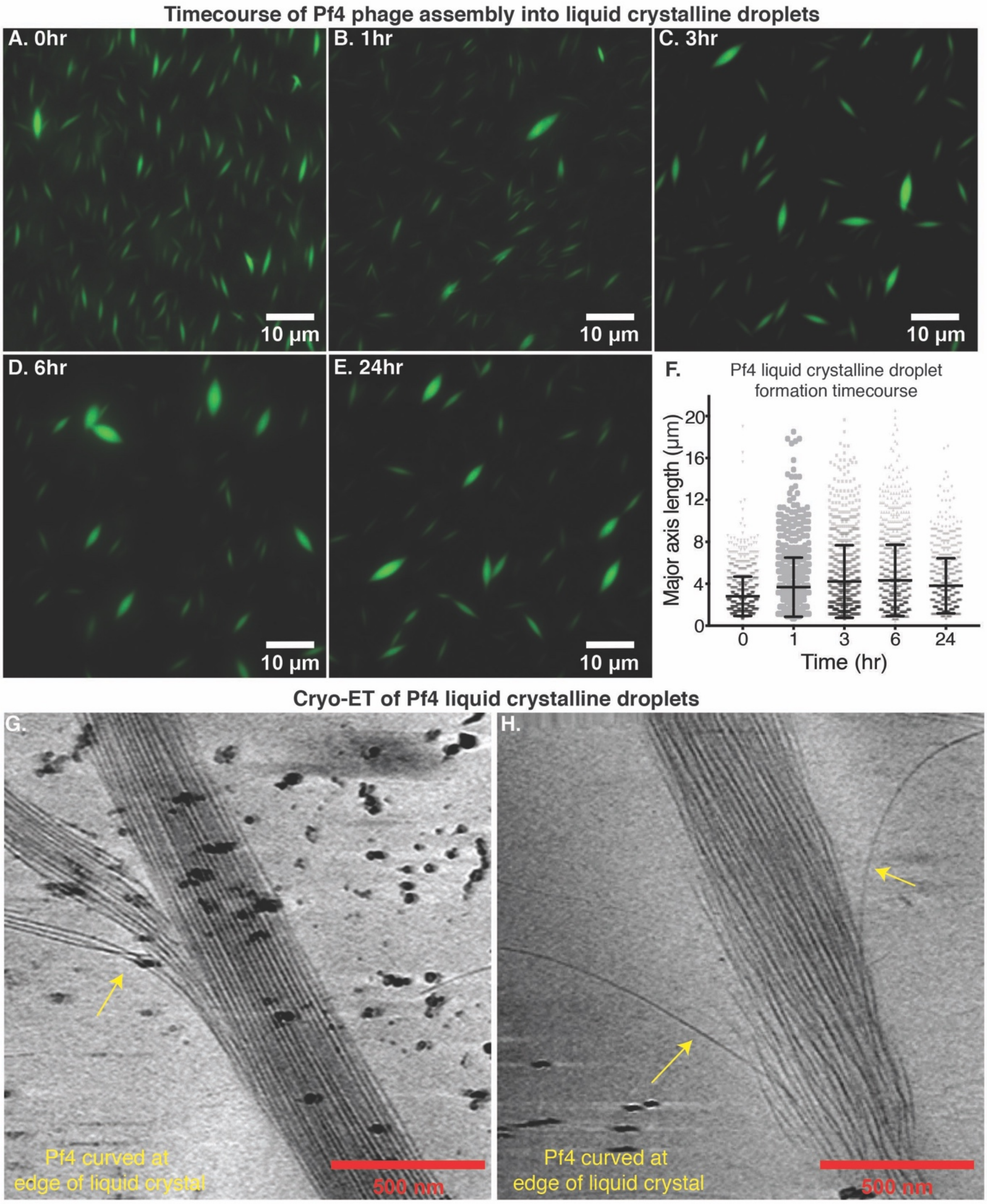

Timecourse of Pf4 phage assembly into liquid crystalline droplets
A. 0hr
B. 1hr
C. 3hr
D. 6hr
E. 24hr
10 µm
F.
Pf4 liquid crystalline droplet formation timecourse
Major axis length (µm)
Time (hr)
Cryo-ET of Pf4 liquid crystalline droplets
G.
H.
Pf4 curved at edge of liquid crystal
500 nm

Fig. S5. Pf4 filament assembly into liquid crystalline droplets (related to Fig. 3)

(A-E) Fluorescence microscopy images of Pf4 liquid crystalline droplets at various time points after mixing A488 labelled-Pf4 (1mg/ml) with sodium alginate (10 mg/ml) in a 1:1 (v/v) ratio. Tactoids were identified by image thresholding and the major axis length of each spindle was measured using Fiji. Small spindle-shaped Pf4 liquid crystalline droplets formed instantly after mixing, panel A) 0 hour (major axis length 2.81 ± 1.87 μm, n = 1280) and grew over time. Panel B) 1 hour after mixing (3.68 ± 2.83 μm, n = 973), panel C) 3 hours after mixing (4.22 ± 3.46 μm, n = 1082), before plateauing in size at panel D) 6 hours after mixing (4.32 ± 3.40 μm, n = 1286). Panel E) 24 hours after mixing Pf4 liquid crystalline droplets were slightly smaller than at 6 hours (3.81 ± 2.63 μm, n = 853). All fluorescence images were background subtracted. (F) Scatter plot showing distribution of droplet major axis lengths (Y-axis) at indicated time points after mixing of components (X-axis). Mean values are shown and the error bars denote standard deviation. (G-H) Cryo-ET of Pf4 liquid crystalline droplets (protein density black) shows longitudinal alignment of Pf4 filaments with the spindle axis and the presence of curved filaments at the spindle edges (indicated by yellow arrows).

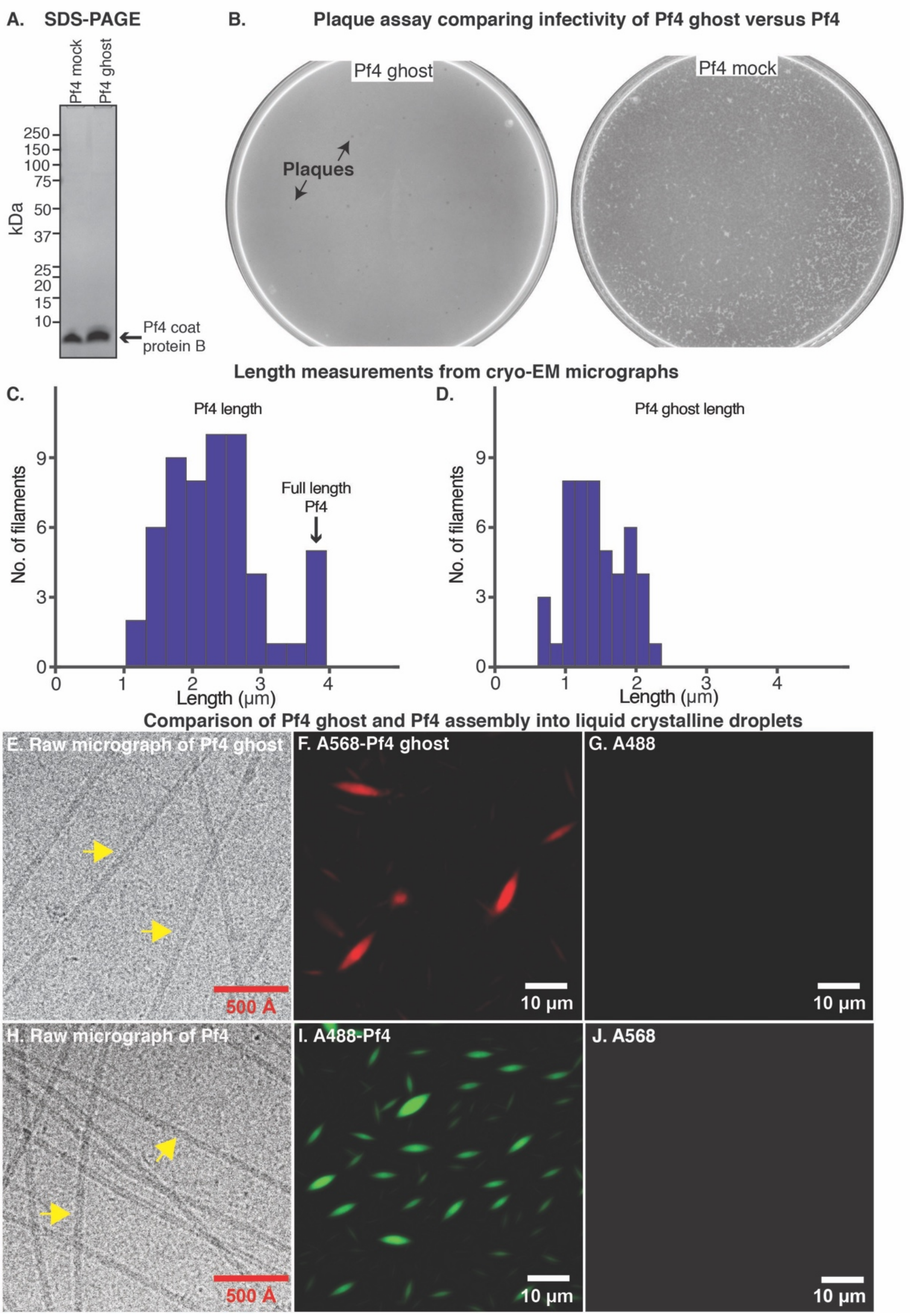
A. SDS-PAGE
Pf4 mock
Pf4 ghost
250
150
100
75
50
37
25
20
15
10
kDa
Pf4 coat protein B
B. Plaque assay comparing infectivity of Pf4 ghost versus Pf4
Pf4 ghost
Pf4 mock
Plaques
Length measurements from cryo-EM micrographs
C.
Pf4 length
Full length Pf4
No. of filaments
Length (μm)
D.
Pf4 ghost length
No. of filaments
Length (μm)
Comparison of Pf4 ghost and Pf4 assembly into liquid crystalline droplets
E. Raw micrograph of Pf4 ghost
500 Å
F. A568-Pf4 ghost
10 μm
G. A488
10 μm
H. Raw micrograph of Pf4
500 Å
I. A488-Pf4
10 μm
J. A568
10 μm

Fig. S6. Preparation of Pf4 ghosts with ssDNA removed (related to Figs. 3 and 4)

(A) SDS-PAGE followed by Coomassie staining of Pf4 mock (ssDNA not extracted) and Pf4 ghost (ssDNA extracted) samples confirms equivalent amounts of Pf4 CoaB protein in both. (B) Plaque assay to measure infectivity of Pf4 ghost and Pf4 mock on PAO1 cells. Left, PAO1 plates infected with Pf4 ghosts show very few plaques formed in a lawn of PAO1 cells (2 x 10^3 pfu/ml). Right, PAO1 plates infected with equivalent amount of Pf4 with ssDNA shows multiple plaques in the lawn of PAO1 (1 x 10^{12} pfu/ml). Pf4 ghosts are several orders of magnitude less infective than native Pf4 with ssDNA, indicating the majority of Pf4 ghost filaments have had the ssDNA genome removed and have been rendered inert. (C-D) Histogram of lengths of native Pf4 and Pf4 ghosts measured from cryo-EM micrographs with counts of filaments (Y-axis) plotted against length of filaments in microns (µm) (X-axis). Full length Pf4 with intact ssDNA genome indicated by the arrow (E) Cryo-EM image of Pf4 ghosts shows presence of filaments (arrows), showing that the removal of ssDNA did not disrupt the filamentous nature of the Pf4 capsid. (F-G) A568-labelled Pf4 (1 mg/ml) mixed 1:1 (v/v) with sodium alginate (10 mg/ml) form spindle-shaped liquid crystalline droplets fluorescent in the A568 channel (panel F) with little fluorescence signal in the A488 channel (panel G). (H) Cryo-EM image of Pf4 with ssDNA shows presence of filaments with the same morphology as Pf4 ghosts (arrows). (I-J) A488-labelled Pf4 (1 mg/ml) mixed 1:1 (v/v) with sodium alginate (10 mg/ml) form spindle-shaped liquid crystalline droplets fluorescent in the A488 channel (panel I) with little fluorescence signal in the A568 channel (panel J).

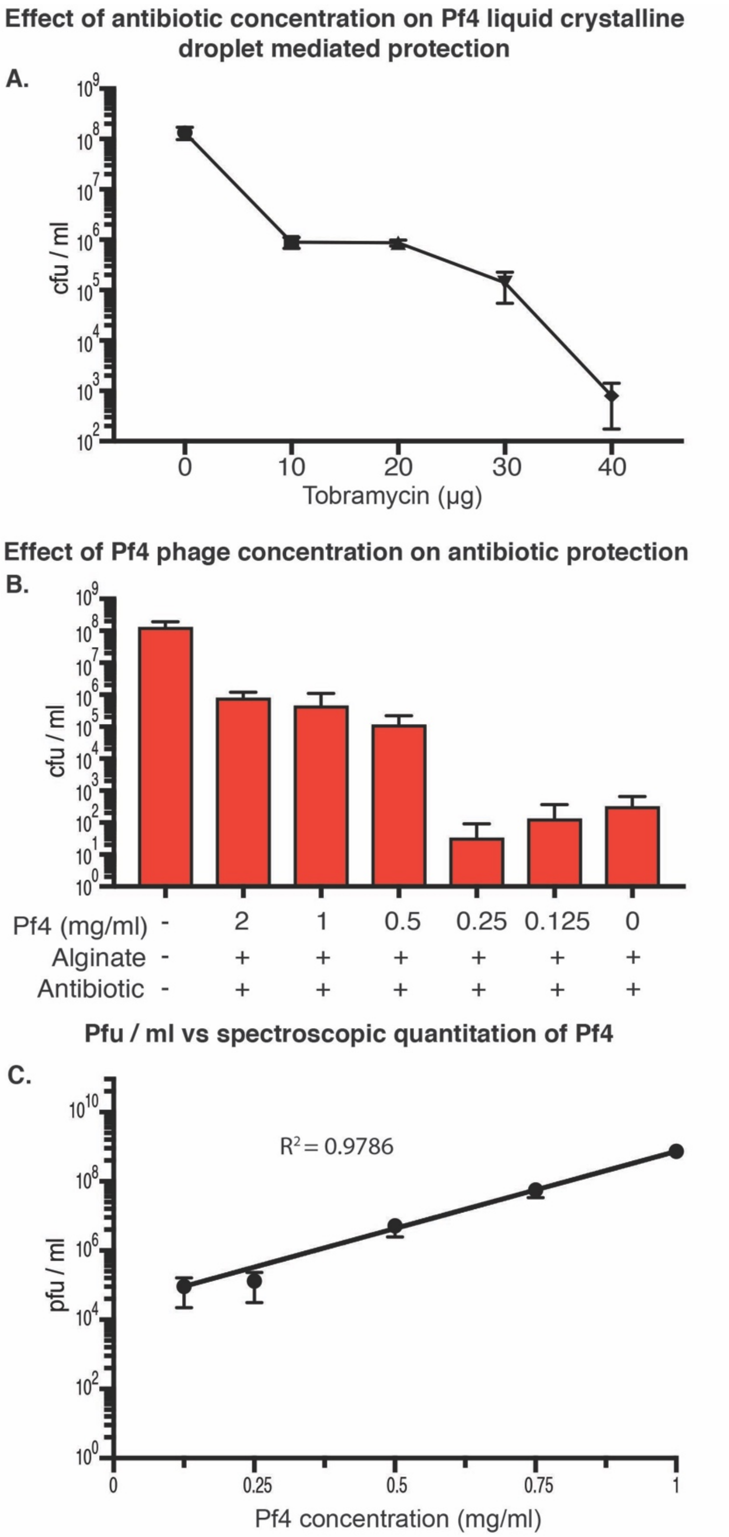
Effect of antibiotic concentration on Pf4 liquid crystalline droplet mediated protection
A.
cfu / ml
Tobramycin (µg)
0
10
20
30
40
Effect of Pf4 phage concentration on antibiotic protection
B.
cfu / ml
Pf4 (mg/ml) - 2 1 0.5 0.25 0.125 0
Alginate - + + + + + +
Antibiotic - + + + + + +
Pfu / ml vs spectroscopic quantitation of Pf4
C.
pfu / ml
R2 = 0.9786
Pf4 concentration (mg/ml)
0
0.25
0.5
0.75
1

Fig. S7 Effect of antibiotic and Pf4 concentration on bacterial protection (related to Fig. 4)

(A) Graph shows effect of antibiotic concentration on Pf4 liquid crystalline droplet mediated protection of *P. aeruginosa*. Y-axis denotes cfu/ml of the surviving bacteria, a measure of *P. aeruginosa* culture cell viability in the presence of Pf4 liquid crystals and increasing amounts of tobramycin (X-axis). (B) Graph shows the effect of Pf4 phage concentration on protection of *P. aeruginosa* against tobramycin. Y-axis denotes cfu/ml in the presence of different reagents and a titration of Pf4 phage concentration (X-axis). (C) Graph showing correlation between pfu/ml (Y-axis) and spectroscopic concentration determination in mg/ml (X-axis) of native Pf4. All experiments were performed in triplicate. Values shown are the mean and error bars denote standard deviation.

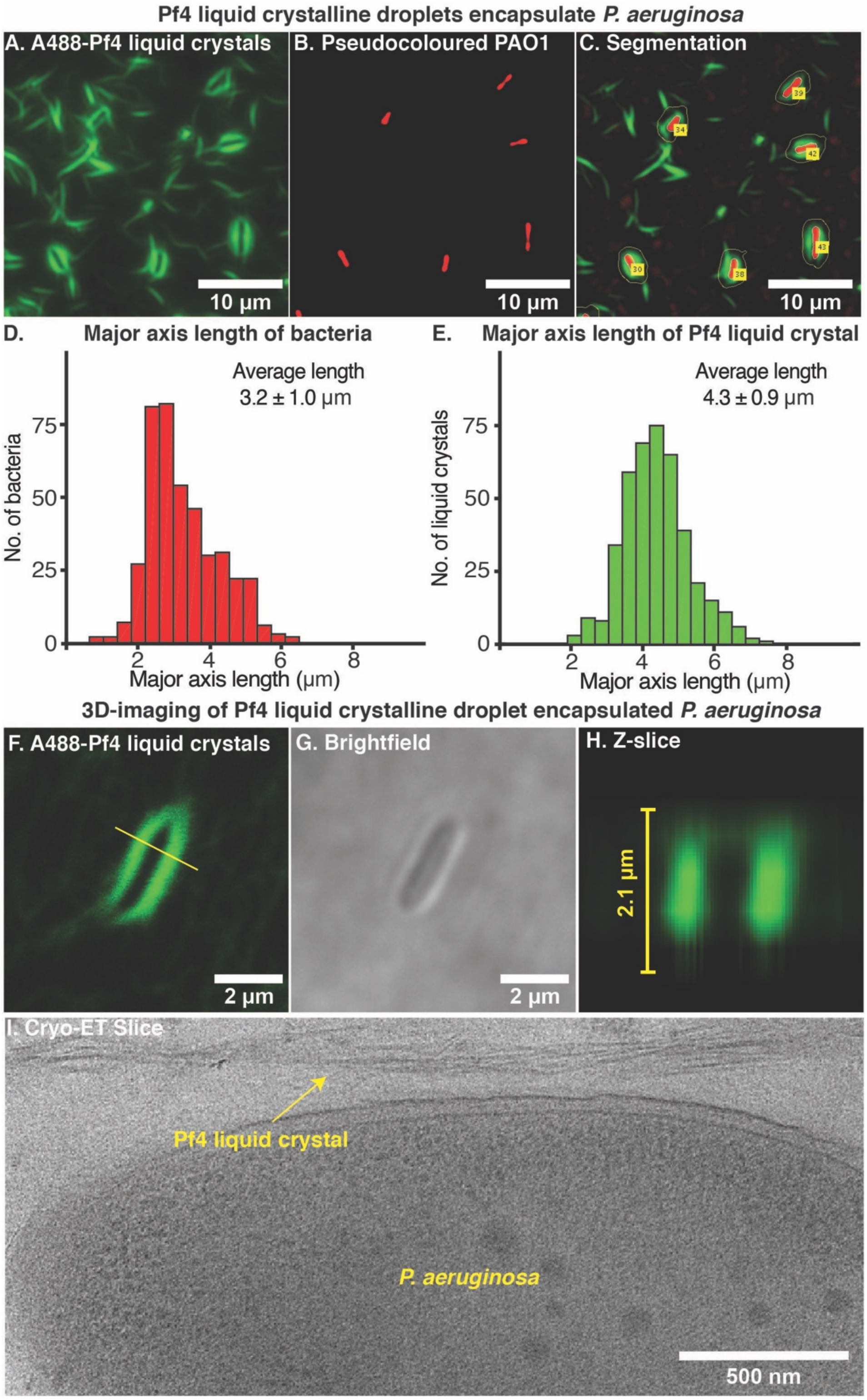
Pf4 liquid crystalline droplets encapsulate P. aeruginosa
A. A488-Pf4 liquid crystals
B. Pseudocoloured PAO1
C. Segmentation
10 µm
10 µm
10 µm
D. Major axis length of bacteria
Average length
3.2 ± 1.0 µm
No. of bacteria
Major axis length (µm)
E. Major axis length of Pf4 liquid crystal
Average length
4.3 ± 0.9 µm
No. of liquid crystals
Major axis length (µm)
3D-imaging of Pf4 liquid crystalline droplet encapsulated P. aeruginosa
F. A488-Pf4 liquid crystals
G. Brightfield
H. Z-slice
2 µm
2 µm
2.1 µm
I. Cryo-ET Slice
Pf4 liquid crystal
P. aeruginosa
500 nm

Fig. S8. Pf4 liquid crystalline droplets encapsulate *P. aeruginosa* cells (related to Fig. 5)

(A-B) Individual channels contributing to merged image shown in Fig. 5A. Panel A) A488-labelled Pf4 liquid crystalline droplets and (panel B) brightfield channel with pseudocoloured *P. aeruginosa* cells (red). (C) Example of automated segmentation of intensities corresponding to liquid crystalline droplet and cells. Segmented regions indicated by yellow outlines. (D) Histogram of the major axis length of segmented bacteria. (E) Histogram of major axis length of bacteria associated liquid crystalline droplets. Length of liquid crystalline droplets (4.3±0.9 µm) were on average 1.1 µm longer than bacteria (3.2±1.0 µm, n = 417). (F-H) Three-dimensional confocal imaging of a *P. aeruginosa* cell encapsulated by a Pf4 liquid crystalline droplet. Panel F) Green channel image showing A488-labelled Pf4 liquid crystalline droplet, panel G) Corresponding brightfield image of encapsulated *P. aeruginosa* cell and panel H) Z-slice through a region indicated on panel F) by yellow line. Even though the liquid crystalline droplet is slightly flattened in the cover slip – agar sandwich, the thickness of the specimen is over 2 µm, making it too thick for imaging using a high-end 300 kV cryo-EM. (I) Cryo-ET slice through a tomogram showing a Pf4 liquid crystalline droplet (tactoid) aligned in close proximity to the bacterial outer membrane. The Pf4 filaments do not touch the bacterial outer membrane, probably due to a meshwork of lipopolysaccharide and other molecules that are known to extend several tens of nanometres outwards from the outer membrane (19).

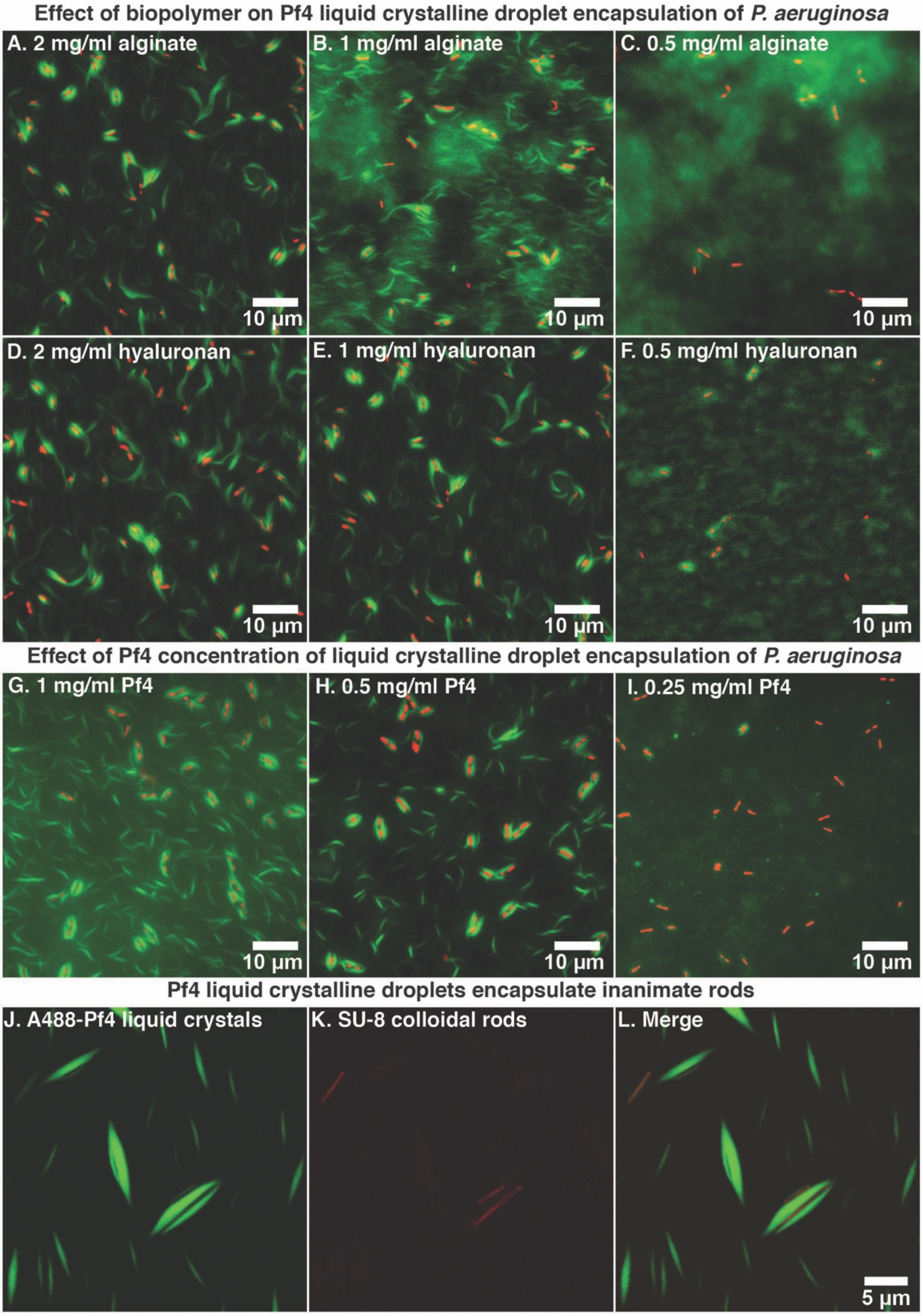
Effect of biopolymer on Pf4 liquid crystalline droplet encapsulation of *P. aeruginosa*
A. 2 mg/ml alginate
B. 1 mg/ml alginate
C. 0.5 mg/ml alginate
D. 2 mg/ml hyaluronan
E. 1 mg/ml hyaluronan
F. 0.5 mg/ml hyaluronan
10 µm
Effect of Pf4 concentration of liquid crystalline droplet encapsulation of *P. aeruginosa*
G. 1 mg/ml Pf4
H. 0.5 mg/ml Pf4
I. 0.25 mg/ml Pf4
Pf4 liquid crystalline droplets encapsulate inanimate rods
J. A488-Pf4 liquid crystals
K. SU-8 colloidal rods
L. Merge
5 µm

Fig. S9. Pf4 liquid crystalline droplet encapsulation of *P. aeruginosa* and inanimate colloidal rods (related to Fig. 5)

(A-C) Effect of alginate concentration on Pf4 liquid crystalline droplet (green) encapsulation of *P. aeruginosa* bacteria (pseudocoloured red). Panel A) 2 mg/ml alginate, panel B) 1 mg/ml alginate and panel C) 0.5 mg/ml alginate. Efficient encapsulation is seen at 1 mg/ml alginate but not 0.5 mg/ml alginate. (D-F) Effect of hyaluronan concentration on Pf4 liquid crystalline droplet (green) encapsulation of *P. aeruginosa* bacteria (pseudocoloured red). Panel D) 2 mg/ml hyaluronan, panel E) 1 mg/ml hyaluronan and panel F) 0.5 mg/ml hyaluronan. Encapsulation is seen with hyaluronan at concentrations as low as 0.5 mg/ml. (G-I) Effect of Pf4 phage concentration on Pf4 liquid crystalline droplet (green) encapsulation of *P. aeruginosa* (pseudocoloured red). Panel G) 1 mg/ml Pf4, panel H) 0.5 mg/ml Pf4 and panel I) 0.25 mg/ml Pf4. Encapsulation is seen at 0.5 mg/ml Pf4 but is no longer apparent at 0.25 mg/ml Pf4. (J-L) Fluorescence microscopic images showing Pf4 liquid crystalline droplets (green) formed around cyanine 3 labelled SU-8 colloidal rods (red) that mimic rod-shaped bacterial cells. This experiment suggests that shape and size complementarity profoundly influences encapsulation.

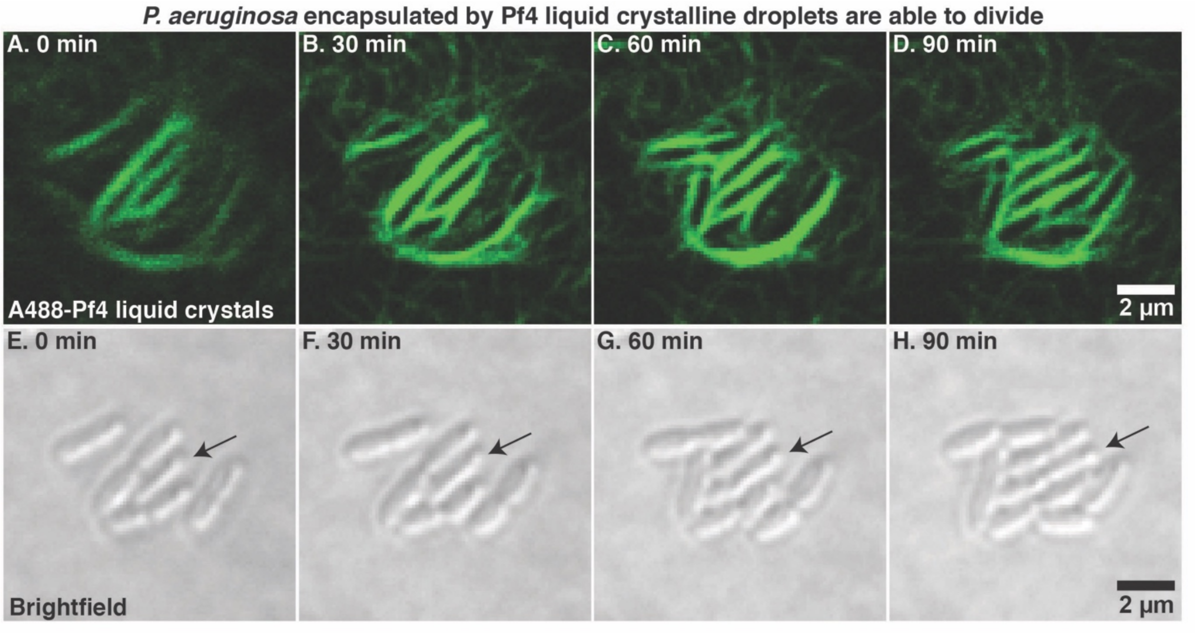
P. aeruginosa encapsulated by Pf4 liquid crystalline droplets are able to divide
A. 0 min
B. 30 min
C. 60 min
D. 90 min
A488-Pf4 liquid crystals
2 µm
E. 0 min
F. 30 min
G. 60 min
H. 90 min
Brightfield
2 µm

Fig. S10 Pf4 liquid crystalline droplet encapsulated *P. aeruginosa* cells are able to divide (related to Fig. 6 and Movie S6)

Time-lapse imaging of Pf4 liquid crystalline droplet encapsulated *P. aeruginosa* cells grown on agar pads at room temperature showing encapsulated cells are able to divide and are therefore metabolically active. (A-D) A488-Pf4 liquid crystalline droplets, panel A) 0 minute, panel B) 30 minute, panel C) 60 minute and panel D) 90 minute time point. (E-H) Brightfield images of *P. aeruginosa* cells, panel E) 0 minute, panel F) 30 minute, panel G) 60 minute and H) 90 minute timepoint. Black arrows indicate dividing cell (see Movie S6).

Table S1: Cryo-EM data collection, refinement and validation statistics

	Pf4 with ssDNA (EMDB-10593) (PDB ID 6TUP)	Pf4 without ssDNA (EMDB-10594) (PDB ID 6TUQ)
**Data collection and processing**		
Magnification	105,000	105,000
Voltage (kV)	300	300
Electron exposure (e^{-}/Å^2)	43	43
Defocus range (μm)	-1 to -3	-1 to -3
Pixel size (Å)	1.38	1.38
Symmetry imposed	Helical	Helical
Initial particle images (no.)	351,381	351,381
Final particle images (no.)	185,002	95,481
Map resolution (Å)	3.2	3.9
FSC threshold	0.143	0.143
**Refinement**		
Initial model used (PDB code)	-	-
Model resolution (Å)	3.3 **(3.1)**	4.2 **(3.9)**
FSC threshold	0.5 **(0.143)**	0.5 **(0.143)**
Map sharpening *B* factor (Å^2)	-68.83	-155.4
Model composition		
Non-hydrogen atoms	16,968	16,422
Protein residues	2,346	2,346
Nucleotides	26	-
B factors (Å^2)		
Protein	93.74	180.97
Nucleotide	205.35	-
R.m.s. deviations		
Bond lengths (Å)	0.012	0.009
Bond angles (°)	1.179	1.004
Validation		
MolProbity score	1.17	1.73
Clashscore	3.77	6.37
Poor rotamers (%)	0.00	3.12
Ramachandran plot		
Favored (%)	100.00	100.00
Allowed (%)	0.00	0.00
Disallowed (%)	0.00	0.00

Supplementary Movie Legends

Movie S1. Cryo-EM structure of Pf4 with ssDNA at 3.2 Å resolution (related to Fig. 1)

The 3.2 Å resolution structure of Pf4 with ssDNA. Cryo-EM density (grey isosurface) and refined atomic coordinates of Pf4 CoaB subunits (ribbon diagrams) are shown. Structure shows the interdigitated arrangement of CoaB α-helices in the phage capsid. Cross-section through the structure reveals that the Pf4 DNA genome is linear and single-stranded (ssDNA). At the end of the video, the cryo-EM density is shown *B*-factor dampened (positive *B*-factor of 50 $Å^2$ applied) to show the fit of the ssDNA built into the density as poly-adenines.

Movie S2. Comparison of Pf4 structures with and without ssDNA (related to Fig. 2)

Comparison of the 3.2 Å resolution Pf4 with ssDNA and 3.9 Å resolution Pf4 without ssDNA structures. Apart from the presence or lack of ssDNA in the inner cavity of the phage, the structures are remarkably similar with the gross morphology of the Pf4 filamentous capsid coat retained in the absence of ssDNA. Comparison of one CoaB subunit from both structures shows no conformational differences.

Movie S3. FRAP experiments on Pf4 liquid crystalline droplets (related to Fig. 3)

A Pf4 liquid crystalline droplet (tactoid) was photobleached multiple times (at 50% laser power) and fluorescence recovery observed (at 1.5% laser power). Photobleaching events are labelled. Video is 160 seconds in length (yellow – high signal, blue – low signal).

Movie S4. Cryo-ET of a Pf4 liquid crystalline droplet (related to Fig. 3)

A video containing sequential Z-slices of a reconstructed electron cryotomogram of a Pf4 liquid crystalline droplet. Individual Pf4 phage filaments are resolved within the droplet, and are longitudinally oriented along the long axis of the spindle.

Movie S5. Cryo-ET of a Pf4 liquid crystalline droplet in association with a *P. aeruginosa* cell (related to Fig. 5)

A video containing sequential Z-slices of a reconstructed electron cryotomogram of a Pf4 liquid crystalline droplet (tactoid) in association with a *P. aeruginosa* cell. The tactoid is in close proximity to the bacterial cell, with some Pf4 filaments wrapping around the pole of the bacteria.

Movie S6. Time-lapse imaging of Pf4 liquid crystalline droplet encapsulated *P. aeruginosa* cells (related to Figs. 6 and S10)

A video showing time-lapse imaging of Pf4 liquid crystalline droplet encapsulated *P. aeruginosa* cells grown on agar pads at room temperature. Images were captured at 10 minute intervals over a 90-minute period. Left, A488-labelled Pf4 liquid crystalline droplets, and right, brightfield of *P. aeruginosa* cells. Video shows that encapsulated cells are metabolically active and able to grow and divide. Pf4 liquid crystalline droplets are able to dynamically re-arrange to accommodate newly divided cells, in line with the deformations seen in Pf4 filaments associated with *P. aeruginosa* cells in cryo-ET.

SI References

1. P. Boulanger, Purification of bacteriophages and SDS-PAGE analysis of phage structural proteins from ghost particles. *Methods Mol. Biol.* **502**, 227-238 (2009).
2. S. Castang, S. L. Dove, Basis for the essentiality of H-NS family members in *Pseudomonas aeruginosa*. *J. Bacteriol.* **194**, 5101-5109 (2012).
3. J. S. Webb, M. Lau, S. Kjelleberg, Bacteriophage and phenotypic variation in *Pseudomonas aeruginosa* biofilm development. *J. Bacteriol.* **186**, 8066-8073 (2004).
4. D. N. Mastronarde, Automated electron microscope tomography using robust prediction of specimen movements. *J. Struct. Biol.* **152**, 36-51 (2005).
5. J. Zivanov *et al.*, New tools for automated high-resolution cryo-EM structure determination in RELION-3. *Elife* **7** (2018).
6. S. Q. Zheng *et al.*, MotionCor2: anisotropic correction of beam-induced motion for improved cryo-electron microscopy. *Nat. Methods* **14**, 331-332 (2017).
7. A. Rohou, N. Grigorieff, CTFFIND4: Fast and accurate defocus estimation from electron micrographs. *J. Struct. Biol.* **192**, 216-221 (2015).
8. R. M. Glaeser, K. Downing, D. DeRosier, W. Chiu, J. Frank, Electron crystallography of biological macromolecules. (2007).
9. S. H. Scheres, S. Chen, Prevention of overfitting in cryo-EM structure determination. *Nat. Methods* **9**, 853-854 (2012).
10. E. F. Pettersen *et al.*, UCSF Chimera--a visualization system for exploratory research and analysis. *J. Comput. Chem.* **25**, 1605-1612 (2004).

11. P. Emsley, B. Lohkamp, W. G. Scott, K. Cowtan, Features and development of Coot. *Acta Crystallogr. D Biol. Crystallogr.* **66**, 486-501 (2010).

12. M. D. Winn *et al.*, Overview of the CCP4 suite and current developments. *Acta Crystallogr. D Biol. Crystallogr.* **67**, 235-242 (2011).

13. P. V. Afonine *et al.*, Real-space refinement in PHENIX for cryo-EM and crystallography. *Acta Crystallogr. D Struct. Biol.* **74**, 531-544 (2018).

14. P. D. Adams *et al.*, PHENIX: a comprehensive Python-based system for macromolecular structure solution. *Acta Crystallogr. D Biol. Crystallogr.* **66**, 213-221 (2010).

15. J. Schindelin *et al.*, Fiji: an open-source platform for biological-image analysis. *Nat. Methods* **9**, 676-682 (2012).

16. R. T. Whitaker, A level-set approach to 3D reconstruction from range data. *Int. J. Comp. Vis.* **29**, 203-231 (1998).

17. J. W. Xu, N. Dayan, A. Goldbourt, Y. Xiang, Cryo-electron microscopy structure of the filamentous bacteriophage IKe. *Proc. Natl. Acad. Sci. USA* **116**, 5493-5498 (2019).

18. C. J. Marzec, L. A. Day, A theory of the symmetries of filamentous bacteriophages. *Biophys. J.* **53**, 425-440 (1988).

19. A. von Kügelgen *et al.*, In Situ Structure of an Intact Lipopolysaccharide-Bound Bacterial Surface Layer. *Cell* **180** (2), 348-358.e15 (2020).
